# Traditional Chinese Medicine Formulae QY305 Reducing Cutaneous Adverse Reaction and Diarrhea by its Nanostructure

**DOI:** 10.1002/advs.202306140

**Published:** 2023-12-03

**Authors:** Ya‐Li Zhang, Ya‐Lei Wang, Ke Yan, Haiyan Li, Xinyu Zhang, Julien Milon Essola, Chengcheng Ding, Kexin Chang, Guangchao Qing, Fuxue Zhang, Yan Tan, Tiantian Peng, Xu Wang, Miao Jiang, Xing‐Jie Liang, Qian Hua

**Affiliations:** ^1^ School of Life Sciences School of Traditional Chinese Medicine Beijing University of Chinese Medicine Beijing 102488 China; ^2^ CAS Center for Excellence in Nanoscience CAS Key Laboratory for Biomedical Effects of Nanomaterials and Nanosafety Chinese Academy of Sciences and National Center for Nanoscience and Technology of China Beijing 100190 China

**Keywords:** cutaneous adverse reaction and diarrhea, nanostructure, pharmacodynamic basis, QY305, traditional Chinese medicine

## Abstract

Traditional Chinese medicine (TCM) is widely used in clinical practice, including skin and gastrointestinal diseases. Here, a potential TCM QY305 (T‐QY305) is reported that can modulate the recruitment of neutrophil in skin and colon tissue thus reducing cutaneous adverse reaction and diarrhea induced by epidermal growth factor receptor inhibitors (EGFRIs). On another hand, the T‐QY305 formula, through regulating neutrophil recruitment features would highlight the presence of N‐QY305, a subunit nanostructure contained in T‐QY305, and confirm its role as potentially being the biomaterial conferring to T‐QY305 its pharmacodynamic features. Here, the clinical records of two patients are analyzed expressing cutaneous adverse reaction and demonstrate positive effect of T‐QY305 on the simultaneous inhibition of both cutaneous adverse reaction and diarrhea in animal models. The satisfying results obtained from T‐QY305, lead to further process to the isolation of N‐QY305 from T‐QY305, in order to demonstrate that the potency of T‐QY305 originates from the nanostructure N‐QY305. Compared to T‐QY305, N‐QY305 exhibits higher potency upon reducing adverse reactions. The data represent a promising candidate for reducing cutaneous adverse reaction and diarrhea, meanwhile proposing a new strategy to highlight the presence of nanostructures being the “*King*” of Chinese medicine formula as the pharmacodynamic basis.

## Graphical abstract

Chinese medicine formula T‐QY305 can reduce cutaneous adverse reaction and diarrhea induced by EGFRIs both in human and animals, which corresponds to “*Jun*”‐“*Chen*”‐“*Zuo*”‐“*Shi*” TCM theory. Meanwhile a new strategy is proposed to highlight the presence of nanostructure N‐QY305 being the “*King*” of Chinese medicine formula as the pharmacodynamic basis.

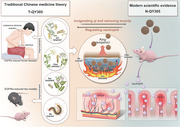

## 1 Introduction

Anticancer treatments, such as chemotherapies and targeted therapy, contribute to improving response rate and progression‐free survival of cancer patients.^[^ ^1^ ^]^ However, the previously cited therapeutic strategies are usually correlated with the development of resistance mechanisms and the occurrence of adverse reactions.^[^ ^2^ ^]^ Here, we focus on adverse reactions induced by anticancer drugs. Among cancer‐targeted therapy, epidermal growth factor receptor inhibitors (EGFRIs) are widely used in clinical practice to treat cancer patients harboring activating mutations in EGFR, especially for non‐small cell lung cancer, breast cancer, colorectal carcinoma.^[^ ^3^ ^]^ With the applications of EGFRIs, multiple adverse reactions are present in several patients, in which cutaneous adverse reaction (including rashes, xerosis, pruritus, paronychia, et al.) and diarrhea are the most common adverse effects.^[^ ^4^ ^]^ During the cancer treatment, a severe occurrence of cutaneous adverse reaction and diarrhea may lead to the temporary termination of anticancer therapy meanwhile drastically affecting the quality of life of patients. These parameters are highly unfavorable for cancer treatment and progression‐free survival.^[^ ^5^ ^]^ Thus, prevention and appropriate management of known toxicities with beneficial agents can be highly effective for supporting the whole cancer treatment. However, emollients or antibiotics (e.g., minocycline) for cutaneous adverse reaction and oral rehydration salts or opioid receptor agonists (e.g., loperamide) for diarrhea frequently recommended by guidelines,^[^ ^6^ ^]^ do not bring satisfactory outcomes.^[^ ^7^ ^]^ Therefore, developing novel therapies for alleviating cutaneous adverse reaction and diarrhea of EGFRIs treatment is urgently needed.

Traditional Chinese medicine (TCM), whose unique theoretical system in ancient China can be traced back 2000 years earlier, has been clinically and scientifically proven effective for treating multiple diseases, including antineoplastic drug‐related adverse reactions.^[^ ^8^ ^]^ The long history of TCM clinical practices is associated with a significant accumulation of a considerable number of Chinese medicine formulas that exhibit both efficacy and safety in vivo, which were formulated based on the TCM theory of “*Jun*” (emperor)‐“*Chen*” (minister)‐“*Zuo*” (assistant)‐“*Shi*” (courier). The conception of this theory refers to the hierarchy of the executive power in the ancient Chinese kingdom. Indeed, in the TCM formula, “‘*Jun*”’ or herb of the monarch achieves the capital task of the treatment, consisting of neutralizing the main symptoms of the disease, while being supported by “*Chen*”, the minister herb, aims to strengthen the defense mechanism triggered by the action of “*Jun*” herb. Then, comes “*Zuo*” or herb of the assistant, which deals with the side damages brought out by “*Jun*” or “*Chen*”, but can also target subordinate diseases or counterbalance the physiological mechanism of the monarch herb, by exhibiting an opposite effect if needed. Last but not least is “*Shi*” or conveyer herb, which literally plays the role of a guide for other molecules, especially when it reaches specific tissues or organs, where the source of the disease is known to be located. Additionally, “*Shi*” can help to harmonize drugs in a formula to eliminate pathogens. Thus, TCM‐based therapy offers the possibility of employing multi‐component drugs and acting on multi‐target, which may be used for clinical trials all over the world.^[^ ^9^ ^]^ Similarly, our study focuses on a Chinese medicine formula T‐QY305, also known as *Qi Yin San Liang San* Decoction, that has been widely used in clinical practice for skin and gastrointestinal diseases. T‐QY305 consists of five herbs: *Astragalus membranaceus* 30 g, *Lonicera japonica* 30 g, *Angelica sinensis* 30 g, *Licorice* 10 g, and *centipede* 1 g. Our preliminary studies found that T‐QY305 could improve EGFRIs‐induced cutaneous adverse reaction and diarrhea in clinical and animal studies.^[^ ^10^ ^]^ However, the pharmacodynamic basis of T‐QY305 reducing EGFRIs‐induced cutaneous adverse reaction and diarrhea remains unknown.

**Scheme 1:**
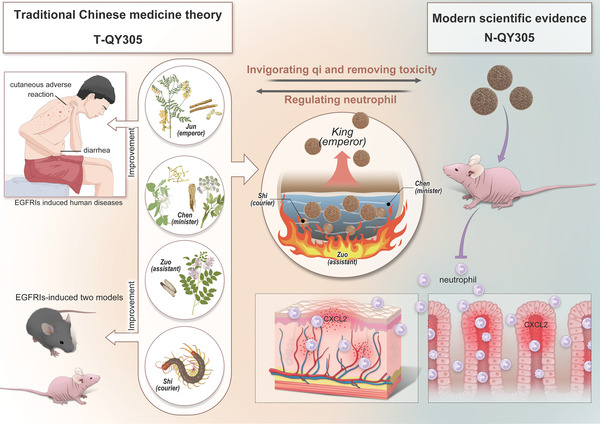
Schematic illustrations of the efficient nanoscale materials from Chinese medicine formula T‐QY305 on reducing cutaneous adverse reaction and diarrhea. The left side of schematic illustrations show that T‐QY305 could reduce cutaneous adverse reaction and diarrhea induced by EGFRIs both in human and animals, which corresponds to “*Jun*”‐“*Chen*”‐“*Zuo*”‐“*Shi*” TCM theory : “*Jun*”‐*Astragalus membranaceus*, “*Chen*”‐*Lonicera japonica*/*Angelica sinensis*, “*Zuo*”‐*Licorice*, “*Shi*”‐*centipede*. The right side of schematic illustrations reveal that nanostructure N‐QY305, extracted from T‐QY305, shows a more powerful effect than T‐QY305, mainly through modulating the infiltration of chemokines and recruitment of neutrophil. It seems to imply that the decoction process of T‐QY305 generates a new “*Jun*” as the “*King*”, namely N‐QY305; the decoction worked as “*Chen*”, the boiling process worked as “*Zuo*” and other components except for N‐QY305 worked as “*Shi*”. Overall, the pharmacodynamic basis of Chinese medicine formula may be related to the nanostructure.

Due to Chinese medicine's unique multi‐component and multi‐target efficacy, it is difficult to clarify its pharmacodynamic basis and effective mechanisms.^[^ ^11^ ^]^ The rise of nanotechnology has allowed its involvement in many research fields, wherein it is widely used in diagnosis, imaging, drug delivery, and other biomedical fields, which are closely related and are even evolving toward integration and convergence to TCM.^[^ ^12^ ^]^ Indeed, self‐assembled nanostructures have been reported to be present in certain Chinese medicine formula and demonstrated to enhance the biological function,^[^ ^13^ ^]^ a feature inherited from Chinese medicine formula based on the TCM theory of “*Jun*”‐“*Chen*”‐“*Zuo*”‐“*Shi*”. Thus, our study is based on the hypothesis of N‐QY305 being the key element for reducing the adverse reaction induced by EGFRIs. We demonstrate the therapeutic efficacy and mechanism of N‐QY305, to reveal the pharmacodynamic basis of QY305 (**Scheme** **1**).

## 2 Results and Discussion

### 2.1 Effect of T‐QY305 on Cutaneous Adverse Reaction Induced by EGFRIs in Clinical Observation

**Figure 1:**
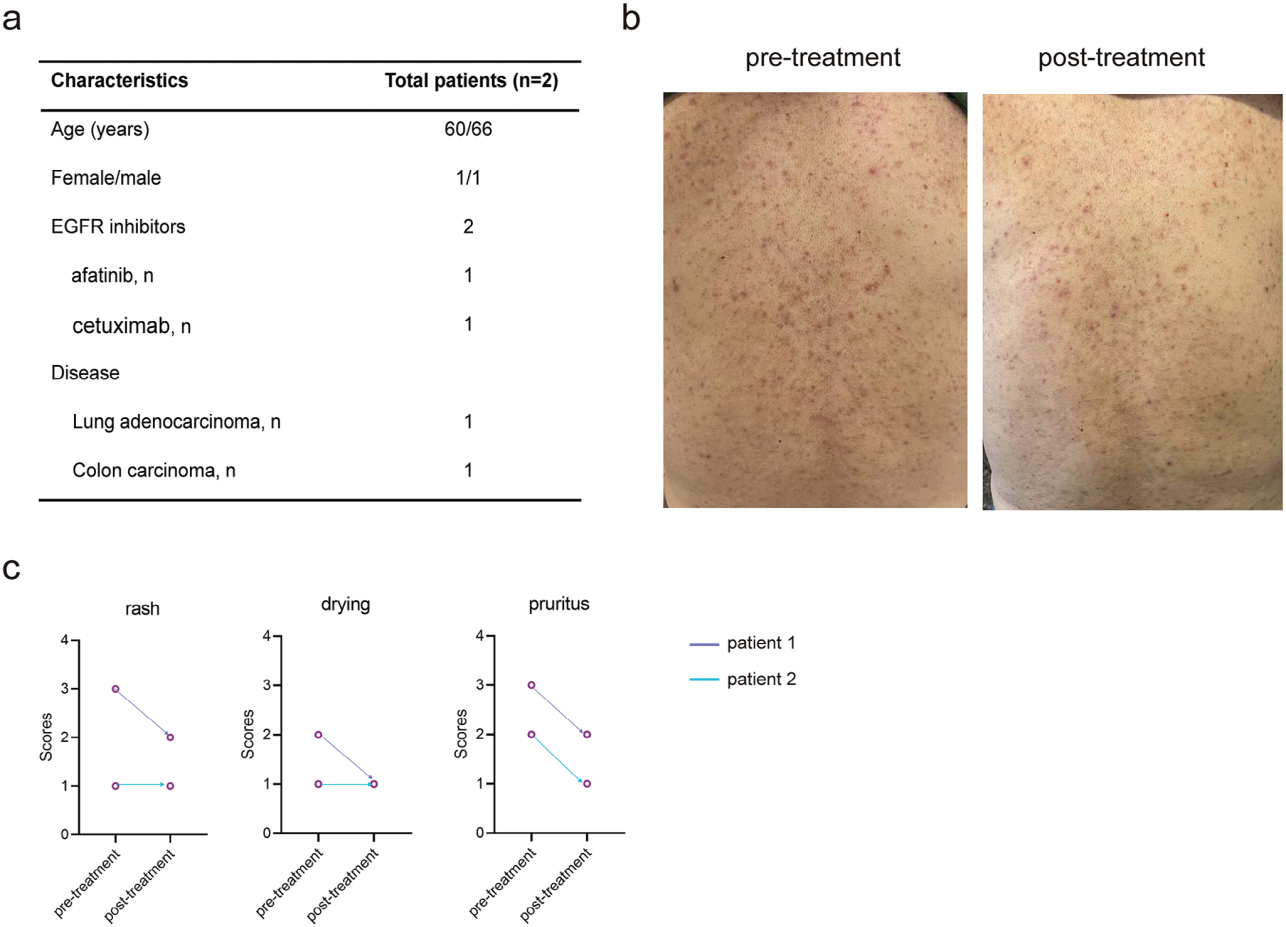
Effect of T‐QY305 on cutaneous adverse reaction induced by EGFRIs in a retrospective clinical study. a) Characteristics of two cancer patients treated with EGFRIs. b) Clinical features of patient 1, pre‐treatment and post‐treatment with oral T‐QY305 daily for 14 days. c) The cutaneous adverse reaction was effectively reduced in the two patients after a daily oral administration of T‐QY305 for 14 days.

EGFRIs has been reported to exhibit a significant anticancer effect. However, a surprising phenomenon following the successful action of anticancer drugs arouses the scientific community's concern, namely, the adverse reactions observed in several patients.^[^ ^1^, ^2^ ^]^ To evaluate the efficacy of T‐QY305 on EGFRIs inducing cutaneous adverse reaction in cancer patients, we carried out clinical observation. Four cancer patients’ information was collected, who were treated with targeted therapy (mainly EGFRIs), occurring cutaneous adverse reaction after a few weeks, and then received T‐QY305 treatment. The collected data of the study are only related to two patients, the reason being that one did not sign the informed consent for some reason and one lost contact with us, so these two patients’ baseline characteristics are listed in **Figure** **1**a and Tables S1 and S2 (Supporting Information). One of the patients was a male of 66 years and the other was a female of 60 years. They both expressed cutaneous adverse reaction and then received T‐QY305 treatment, through oral administration twice a day for 14 days. The study revealed that the rash, drying, and pruritus of the two patients had varying degrees of symptomatic relief with T‐QY305 treatment (Figure 1b,c; Figure S1, Supporting Information). Importantly, no other T‐QY305 relevant adverse reactions were found. Actually, it could reduce diarrhea in several patients; however, the clinical data could not be monitored for some reasons.

### 2.2 T‐QY305 Reduced the Cutaneous Adverse Reaction and Diarrhea Induced by Gefitinib in balb/c Nude Mice Carrying Tumor

**Figure 2:**
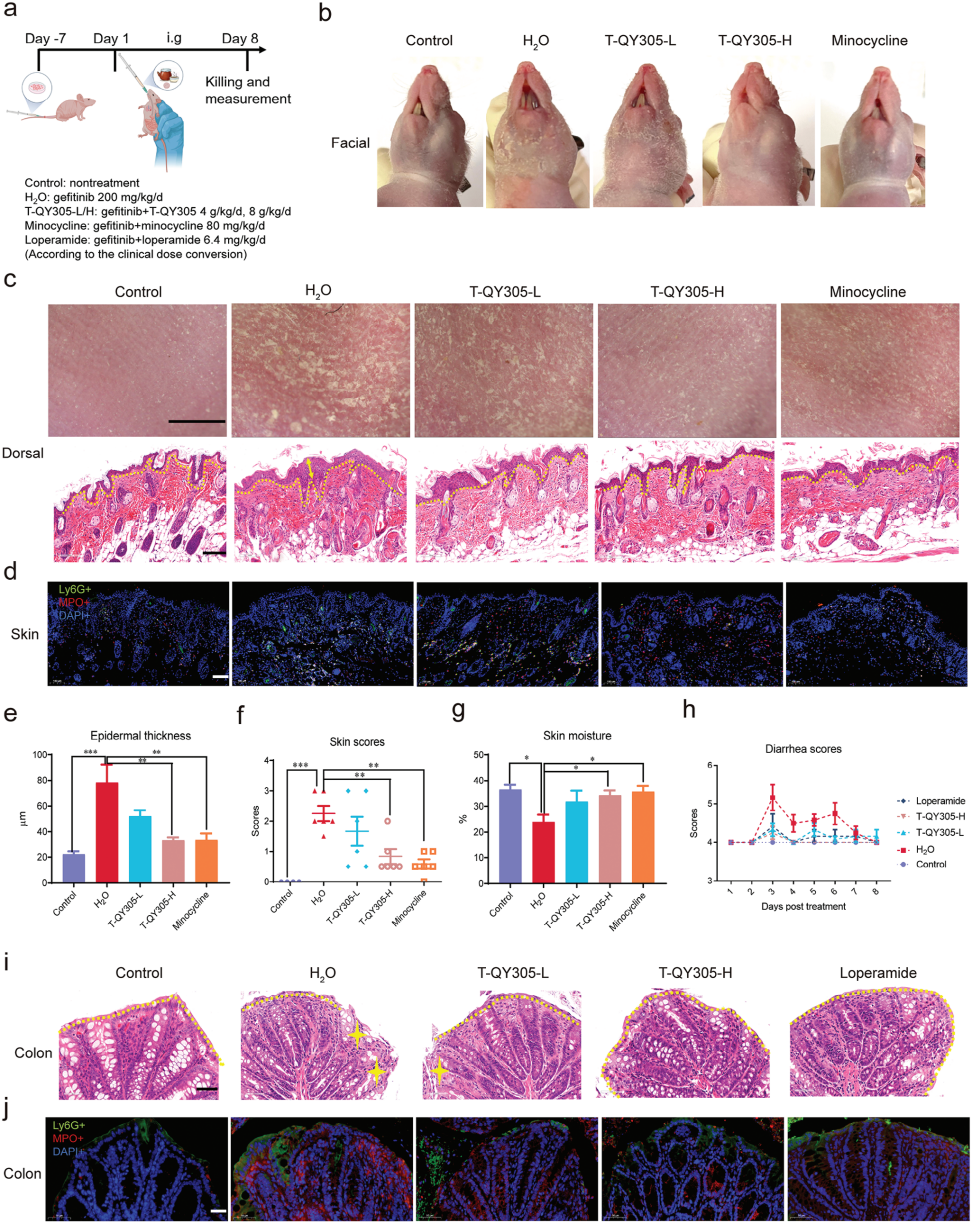
T‐QY305 reduced the gefitinib‐induced cutaneous adverse reaction and diarrhea in balb/c nude mice carrying tumor. a) Experimental timeline for treatment of cutaneous adverse reaction and diarrhea induced by gefitinib in balb/c nude mice carrying tumor (n: control group = 4, other groups = 6). b) The phenotype of facial skin captured by mobile devices (n = 3). c) Top row: the phenotype of dorsal skin captured by skin detector (n = 3), scale bar = 0.6 cm. Lower row: the thickness of the skin epidermis detected by H&E staining (n = 3), scale bar = 100 µm. d) Immunofluorescence staining for Ly6G^+^ (green) and MPO^+^ (red) with DAPI (blue) of skin tissue from balb/c nude mice (n = 3), scale bar = 100 µm. e) Statistical results of epidermal thickness of skin tissue (n = 3). f) The scores of skin inflammation were evaluated as referenced, score 0, normal; score 1, quarter of the whole body; score 2, half of the whole body; score 3, greater than or equal to three‐quarters of the whole body (n≥4). g) The skin moisture was assessed by Delfin MoistureMeterEpiD (n≥4). h) The severity of diarrhea was scored daily followed by Bristol Stool Scale: score 4, normal; score 5, mild diarrhea; score 6, moderate diarrhea; score 7, severe diarrhea (n≥4). i) The phenotype of colon tissue detected by H&E staining (n = 3), the stars above the colon represent the damaged area, scale bar = 50 µm. j) Immunofluorescence staining for Ly6G^+^ (green) and MPO^+^ (red) of colon tissue from balb/c nude mice (n = 2), scale bar = 50 µm. Data are presented as means ± SEM, ^*^ *p* <0.05, ^**^ *p* <0.01, ^***^ *p* <0.005, ^****^ *p* <0.001, “^*^” compared with H_2_O group (model).

As described in the introduction, EGFRIs is associated with adverse effects such as cutaneous adverse reaction and diarrhea. In fact, cutaneous adverse reaction and diarrhea induced by EGFRIs may simultaneously appear in a patient.^[^ ^14^ ^]^ For a better stimulation of the clinical course of cutaneous adverse reaction and diarrhea induced by EGFRIs, and further investigate the effect of T‐QY305, we developed an orthotopic non‐small cell lung cancer model in balb/c nude mice (Figures S2–S5, Supporting Information), in which we established cutaneous adverse reaction and diarrhea occurring at the same time. The establishing process of the animal model was described in the supplementary materials section. Next, we tested the efficacy of different dosages of T‐QY305 or positive drugs on gefitinib‐induced cutaneous adverse reaction and diarrhea in balb/c nude mice carrying tumor. As shown in **Figure** **2**a, balb/c nude mice carrying tumor were treated through oral administration with vehicle (control), H_2_O (model), T‐QY305 of two concentrations (low‐dose, T‐QY305‐L 4 g kg^−1^ d^−1^; high‐dose, T‐QY305‐H 8 g kg^−1^ d^−1^, according to the clinical dose conversion) and positive control groups (minocycline 80 mg kg^−1^ d^−1^ for cutaneous adverse reaction or loperamide 6.4 mg kg^−1^ d^−1^ for diarrhea).

For cutaneous adverse reaction effects of T‐QY305, before being euthanized, we collected facial and dorsal skin images of the mice. T‐QY305 treatment significantly protected the skin phenotype of facial and dorsal skin from the eventual damages induced by gefitinib (Figure 2b,c), as well as reduced gefitinib‐induced epidermis thickening and scores of skin inflammation (Figure 2c,e,f). More importantly, T‐QY305 was demonstrated to be as highly efficient as minocycline, regarding its ability to increase skin moisture, an index of skin barrier function (Figure 2g). However, there is no relief for diarrhea under minocycline treatment (Figure S6a, Supporting Information).

For diarrhea effect of T‐QY305, fecal excretion of animals was scored as Bristol Stool Scale.^[^ ^15^ ^]^ It demonstrated significant potency for reducing diarrhea occurrences or severity induced by gefitinib (Figure 2h). In addition, T‐QY305 also reduced the damage to colonic epithelium (Figure 2i). Although loperamide reduced diarrhea occurrence or severity induced by gefitinib, finding the right balance between diarrhea and constipation (Figure S6b, Supporting Information), a side effect of loperamide, is challenging.^[^ ^16^ ^]^

As mentioned in the literature, neutrophil may be essential for skin and colon inflammation.^[^ ^17^ ^]^ To further investigate the effect of T‐QY305 on the recruitment of neutrophil in skin and colon tissue, neutrophil markers (Ly6G^+^MPO^+^) were detected by immunofluorescence. The results of neutrophil immunofluorescence demonstrated a decrease in neutrophil recruitment into skin and colon tissue after treatment with T‐QY305 (Figure 2d,j). Furthermore, all the groups treated with gefitinib highlighted its efficacy in inhibiting tumor growth in balb/c nude mice. Additionally, it is to be mentioned that the combination of gefitinib with T‐QY305 did not affect the anticancer efficacy of gefitinib (Figure S7, Supporting Information). More importantly, no significant toxicity related to the pathology was observed in any major organ system of mice treated with T‐QY305, and no significant weight changes (Figure S8a–c, Supporting Information).

### 2.3 T‐QY305 Reduced the Cutaneous Adverse Reaction and Diarrhea Induced by Gefitinib in BN Rats

**Figure 3:**
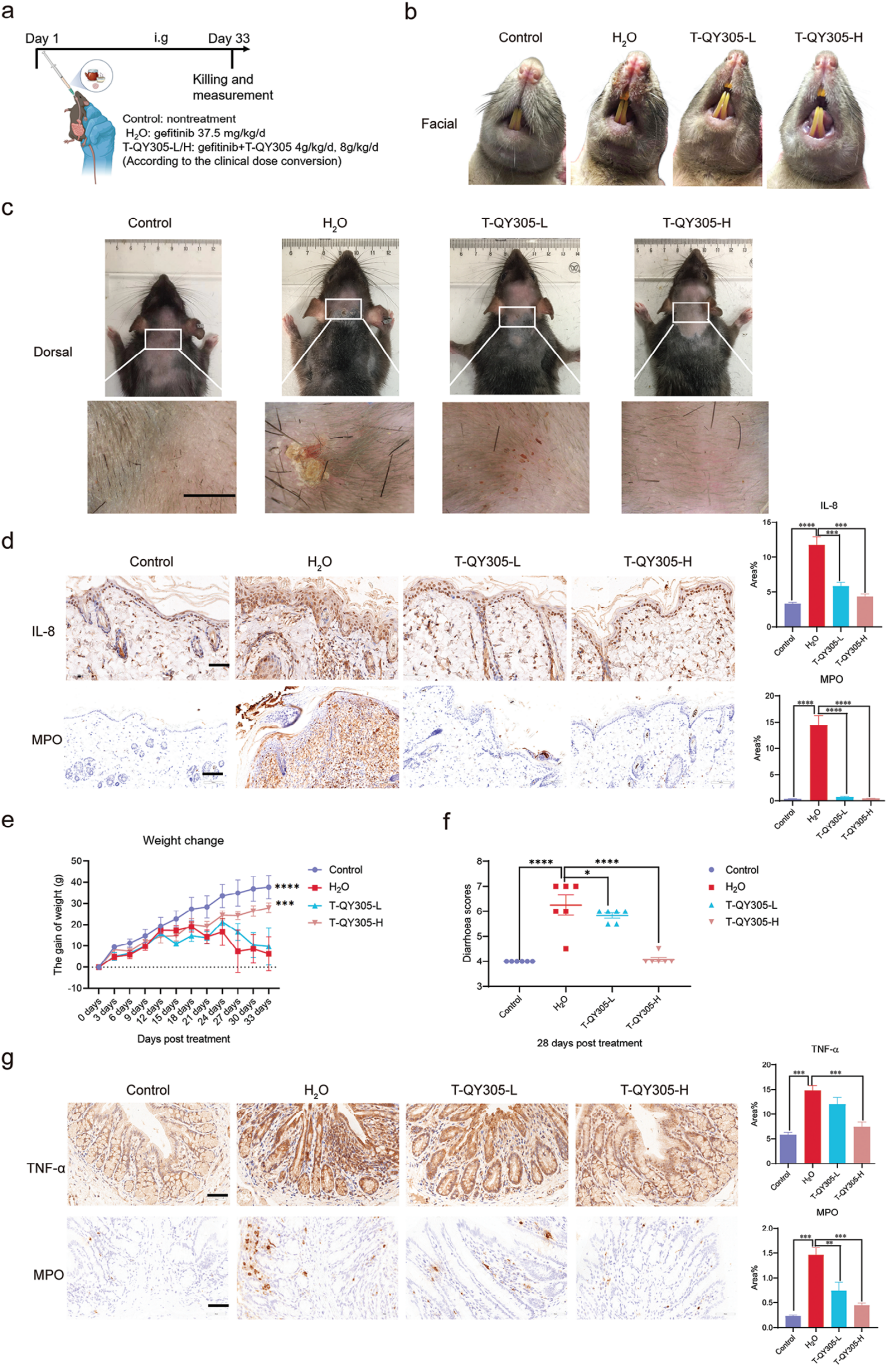
T‐QY305 reduced the cutaneous adverse reaction and diarrhea induced by gefitinib in BN rats. a) Experimental timeline for T‐QY305 treatment of gefitinib‐induced cutaneous adverse reaction and diarrhea in BN rats, n = 6. b) Phenotype of facial skin captured by mobile devices (n = 3). c) Top row: the phenotype of dorsal skin captured by mobile devices (n = 3). Lower row: the phenotype of dorsal skin was captured by skin detector (n = 3), scale bar = 0.6 cm. d) Skin tissue representative immunohistochemistry images of neutrophil chemokine (IL‐8^+^, scale bar = 50 µm, top row, n = 3) and marker of neutrophil recruitment (myeloperoxidase, MPO^+^, scale bar = 100 µm, lower row, n = 3). e) The body weight was measured and recorded every 3 days, which was followed by the display of weight changes (n≥4). f) The severity of diarrhea was scored on day 28 by Bristol Stool Scale (n = 6): score 4, normal; score 5, mild diarrhea; score 6, moderate diarrhea; score 7, severe diarrhea. g) Colon tissue representative immunohistochemistry images of inflammatory cytokine (TNF‐α^+^, scale bar = 50 µm, top row, n = 3) and the marker of neutrophil recruitment (myeloperoxidase, MPO^+^, scale bar = 50 µm, lower row, n = 3). Data are presented as means ± SEM, ^*^ *p* <0.05, ^***^ *p* <0.005, ^****^ *p* <0.001, “^*^” compared with H_2_O group (model).

In order to further clarify the efficacy of T‐QY305, we designed groups of experiments in BN rats to assess whether T‐QY305 could simultaneously decrease cutaneous adverse reaction and diarrhea induced by gefitinib. The BN rats model displaying cutaneous adverse reaction and diarrhea was created similarly to the one designed in the previous study.^[^ ^18^ ^]^ As shown in **Figure** **3**a, BN rats were treated through oral administration with vehicle (control), H_2_O (model), T‐QY305 of two concentrations (low‐dose, T‐QY305‐L 4 g kg^−1^ d^−1^; high‐dose, T‐QY305‐H 8 g kg ^−1^d^−1^, according to the clinical dose conversion).

To analyze the efficacy of T‐QY305 in inhibiting cutaneous adverse reaction, we collected facial and dorsal skin images of all rats before euthanizing them. The results showed that T‐QY305 had a significant potency for the inhibition of inflammatory reactions occurring on the facial and dorsal skin of the rats treated with gefitinib (Figure 3b,c). Also, the results suggested that T‐QY305 reduced gefitinib‐induced epidermis thickening (Figure S9a, Supporting Information). Furthermore, T‐QY305 showed significant efficacy upon gefitinib‐induced phenomenon of inflammatory factors and undesirable inflammatory cell infiltration into the skin tissue. Among them are inflammatory cytokine of IL‐8 (neutrophil chemokine) (Figure 3d, top row) and TNF‐α (Figure S10a, Supporting Information, top row, b, left), along with inflammatory cells neutrophil (MPO^+^) (Figure 3d, lower row) and macrophage (CD68^+^) (Figure S10a, Supporting Information, lower row, b right), which showed a dose dependence and corroborated the analysis completed in the previous reports.^[^ ^19^ ^]^

Regarding diarrhea, T‐QY305 has significantly improved food intake (Figure S9b, Supporting Information), thus reducing the loss of body weight (Figure 3e) induced by gefitinib. During the early 7 days following the administration of gefitinib intervention, the effect of T‐QY305 on diarrhea was not observed (Figure S9c, Supporting Information), however from day 28, two doses of T‐QY305 were able to decrease diarrhea scores induced by gefitinib (Figure 3f). Besides, T‐QY305 also reduced the damage of colonic epithelium (Figure S9d, Supporting Information, top row) and the number of goblet cells induced by gefitinib (Figure S9d, Supporting Information, lower row). At the same time, T‐QY305 reduced gefitinib‐induced colon tissue infiltration of inflammatory cytokine of TNF‐α (Figure 3g, top row) as well as colon tissue recruitment of inflammatory cells of neutrophil (MPO^+^) (Figure 3g, lower row) and macrophages (CD68^+^) (Figure S11a,b, Supporting Information).

### 2.4 Nanostructures from T‐QY305 May Be the Key Components for Efficacy on Adverse Reactions

Recently, the constantly combined nanotechnology with TCM, has solved some application limitations of it, such as enhancing solubility, improving bioavailability, and increasing the targeting and penetration.^[^ ^12^, ^20^ ^]^ It is a promising strategy and provides more possibilities for TCM therapeutic effects.^[^ ^8^, ^21^ ^]^ There have been a number of reports describing nanoparticles existed in some Chinese herbal decoction, which exhibited a stable form for better effects, meaning a new possible mechanism of the action of Chinese medicine formula.^[^ ^13^, ^22^ ^]^ Beyond that, nanostructure also has been used to explain the generation of supramolecular self‐assembly during the boiling process of herbals, which could reveal the pharmacodynamic basis, that actually originates from the integrity of Chinese medicine formula based on the TCM theory of “*Jun*”‐“*Chen*”‐“*Zuo*”‐“*Shi*”.^[^ ^23^ ^]^

**Figure 4:**
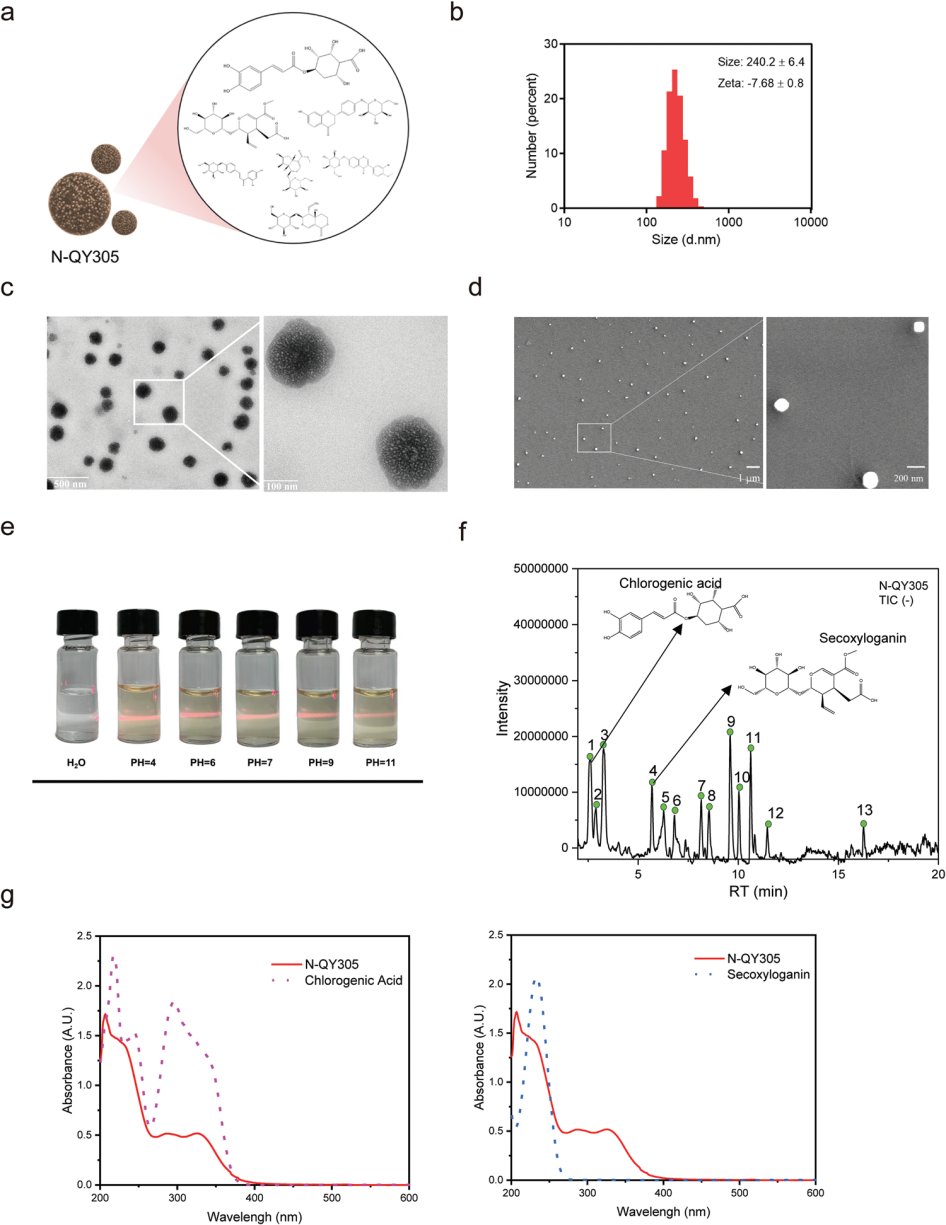
Preparation and characterization of N‐QY305. a,b) N‐QY305 was prepared from T‐QY305 and its size distribution and zeta potential were measured by DLS. c,d) TEM and SEM images of N‐QY305. e) The Tyndall effect of different PH solutions of N‐QY305. f) LC‐MS/MS analysis of N‐QY305. g) UV–vis spectra of chlorogenic acid and secoxyloganin.

As stipulated in the hypothesis of our work, there is a high probability that the employed TCM in our study (T‐QY305) encompasses a nanostructure that constitutes the pharmacodynamic basis of T‐QY305. In this optic, we proceeded to extract the nanoparticle contained in T‐QY305, labeled N‐QY305 (**Figure** **4**a). The extraction of N‐QY305 nanoparticle was followed by its physicochemical characterization, consisting of analyzing its average size, surface charge, morphological features, stability as well as dispersity. The size and zeta potential were respectively estimated to be 240.2 ± 6.4 nm and −7.68 ± 0.8 mV (Figure 4b). Then morphological features of the particle were observed through transmission electron microscopy (TEM) and scanning electron microscope (SEM) imaging and showed that N‐QY305 possesses a regular spherical shape (Figure 4c,d). Further, we proceeded to the analysis of the dispersion feature of N‐QY305 in a medium by observing the light scattering effect of N‐QY305 colloidal particles in a suspension (Tyndall effect), through one hour incubation of N‐QY305 in buffers with different pH values. The Tyndall effect was meticulously observed in the different buffers, in which N‐QY305 was incubated, and a drastic decrease of light scattering by the N‐QY305 colloidal particles was noticed in only a buffer harboring a pH of 11.0. It was suggested that N‐QY305 was relatively stable and may be easier to disassemble under strongly alkaline conditions (Figure 4e).

On the basis of the data enlisted by the available literature and standards (Figure S12a and Table S3, Supporting Information), we were able to identify by Liquid chromatograph mass spectrometer (LC‐MS/MS), 21 major components of T‐QY305 (Figure S12b and Table S4, Supporting Information) and 13 main components of N‐QY305 (Figure 4f; Table S5, Supporting Information). In comparison with other components, chlorogenic acid (peak 1′) and secoxyloganin (peak 4′) were detected at the highest levels in skin tissue, but also in inflamed tissue in comparison with normal tissue (Figure 4f; Figure S13, Supporting Information). Thus, the UV spectra of chlorogenic acid and secoxyloganin were measured, which peaks obtained with chlorogenic acid and secoxyloganin exhibited high similarity with N‐QY305 peaks (Figure 4g). Beyond that, N‐QY305 has the similar stability (similar UV spectra) to T‐QY305 after incubating in simulated gastric fluid (SGF) for 2 h (Figure S14, Supporting Information).

According to the TCM theory, each herb possesses a particular function in the curing mechanism of the disease. Indeed, as a TCM‐based therapeutic, T‐QY305, follows the same path, and each of its herbal components can be classified in a category, namely: “*Jun*”‐ *Astragalus membranaceus*, “*Chen*”‐*Lonicera japonica*/*Angelica sinensis*, “*Zuo*”‐*Licorice*, “*Shi*”‐*centipede*. Precisely due to the “multi‐ingredients” and “multi‐targets” features of Chinese medicine formula,^[^ ^24^ ^]^ the boiling process could generate self‐assembled nanomaterials, which did not require additional carrier and acted as a whole system, making up for the shortcomings of a single small molecule compound.^[^ ^9a^ ^]^ Hence, we suggested that T‐QY305's ability to suppress cutaneous adverse reaction and diarrhea originates from its subunit nanostructure N‐QY305, in which considered N‐QY305 as mediating the “*King*” (“*Jun*”) function in the treatment of adverse reactions, the decoction worked as “*Chen*”, the boiling process worked as “*Zuo*” and other components except for N‐QY305 worked as “*Shi*”. Thus, the next section will aim to analyze the biological mechanisms involved in N‐QY305 inhibition of cutaneous adverse reaction and diarrhea caused by gefitinib, but also to assess its level of efficacy.

### 2.5 N‐QY305 Reduced the Cutaneous Adverse Reaction and Diarrhea Induced by Gefitinib Mainly by Regulating Neutrophil Infiltration in Skin and Colon Tissue

**Figure 5:**
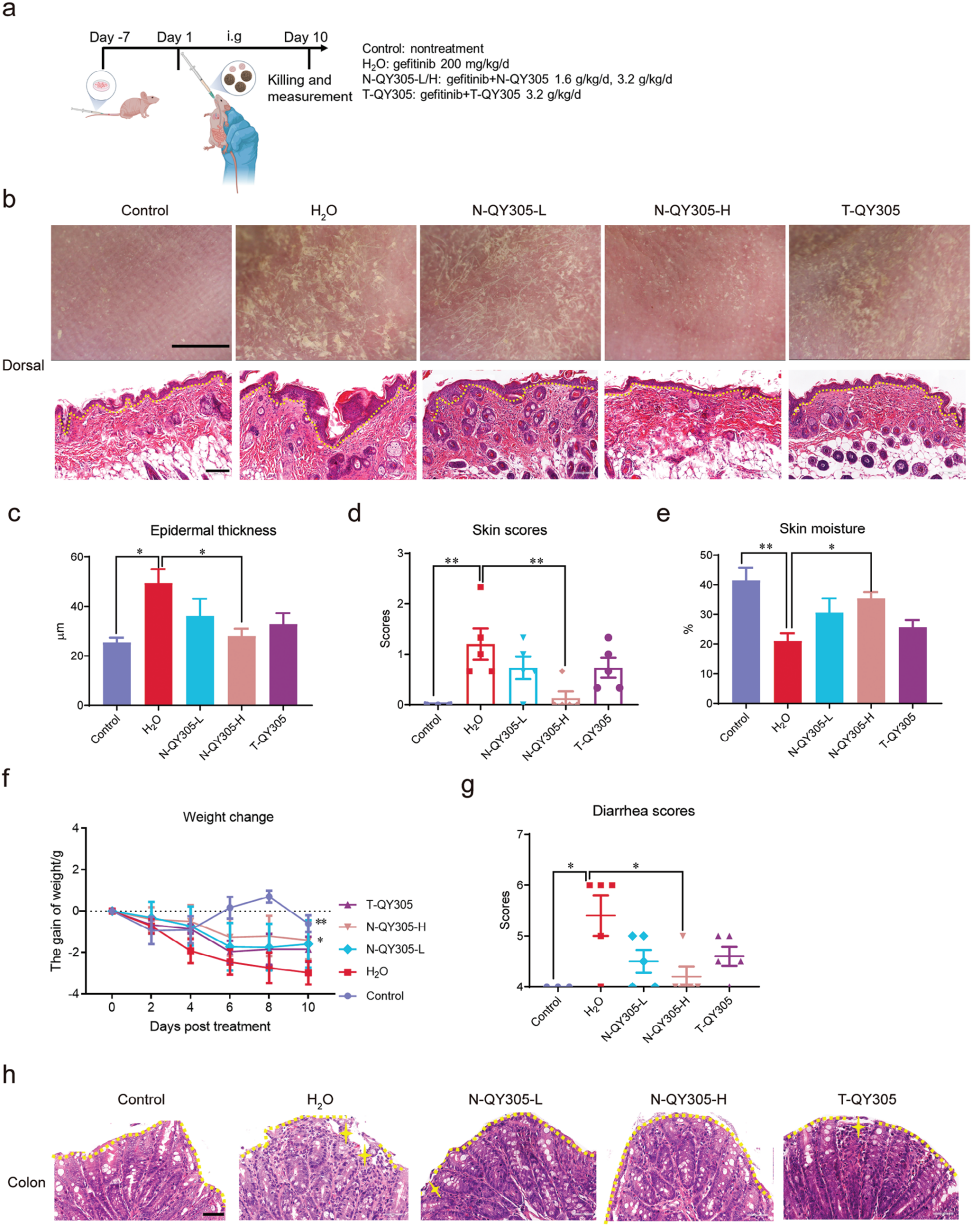
N‐QY305 reduced gefitinib‐induced cutaneous adverse reaction and diarrhea in balb/c nude mice carrying tumor. a) Experimental timeline for N‐QY305 treatment of cutaneous adverse reaction and diarrhea induced by gefitinib in balb/c nude mice carrying tumor. b) Top row: Phenotype of dorsal skin captured by skin detector (n = 3), scale bar = 0.6 cm. Lower row: Thickness of the skin epidermis detected by H&E staining (n = 3), scale bar = 100 µm. c) Statistical results of epidermal thickness of skin tissue (n = 3). d) The scores of skin inflammation were evaluated as referred, score 0, normal; score 1, quarter of the whole body; score 2, half of the whole body; score 3, greater than or equal to three‐quarters of the whole body (n≥4). e) The skin moisture was assessed by Delfin MoistureMeterEpiD (n≥4). f) The body weight was measured and recorded every 2 days (n≥4). g) The severity of diarrhea was scored daily followed by Bristol Stool Scale: score 4, normal; score 5, mild diarrhea; score 6, moderate diarrhea; score 7, severe diarrhea. h) Phenotype of colon tissue detected by H&E staining (n = 3), the stars above the colon represent the damaged epithelial cells, scale bar = 50 µm. Data are presented as means ± SEM, ^*^ *p* <0.05, ^**^ *p* <0.01, ^***^ *p* <0.005, “^*^” compared with H_2_O group (model).

We tested the efficacy of different dosages of N‐QY305 on gefitinib‐induced cutaneous adverse reaction and diarrhea in balb/c nude mice carrying tumor. As shown in **Figure** **5**a, balb/c nude mice carrying tumor were treated by oral administration with vehicle (control), H_2_O (model), N‐QY305 of two concentrations (low‐dose, N‐QY305‐L 1.6 g kg ^−1^d^−1^; high‐dose, N‐QY305‐H 3.2 g kg ^−1^d^−1^, according to the clinical dose conversion) and T‐QY305 3.2 g kg ^−1^d^−1^ group. To analyze the cutaneous adverse reaction effect of N‐QY305, we collected dorsal skin images of the mice before euthanizing them. The results showed that N‐QY305 could also improve the phenotype of dorsal skin (Figure 5b), decrease the scores of skin inflammation (Figure 5c) and reduce gefitinib‐induced epidermis thickening (Figure 5b,d). Beyond that, the index of skin barrier function showed an increasing trend (Figure 5e). For diarrhea effect of N‐QY305, it could decrease the loss of body weight and reduce diarrhea scores induced by gefitinib (Figure 5f,g). In addition, N‐QY305 was able to decrease the damage to colonic epithelium as well (Figure 5h). And similar to the results obtained in Figure S6 (Supporting Information), N‐QY305 had no negative impact on gefitinib anticancer effect in balb/c nude mice (Figure S15, Supporting Information). The toxicity of the N‐QY305 was further observed, and we found no significant toxicity in weight change, AST, ALT, BUN, UA and CRE levels in blood plasma and any major organ system of N‐QY305 treated mice (Figure S16, Supporting Information). The collected results allowed us to conclude that N‐QY305 could not only effectively reduce the cutaneous adverse reaction and diarrhea induced by gefitinib, but also exhibited higher efficacy than T‐QY305 at the same dose.

**Figure 6:**
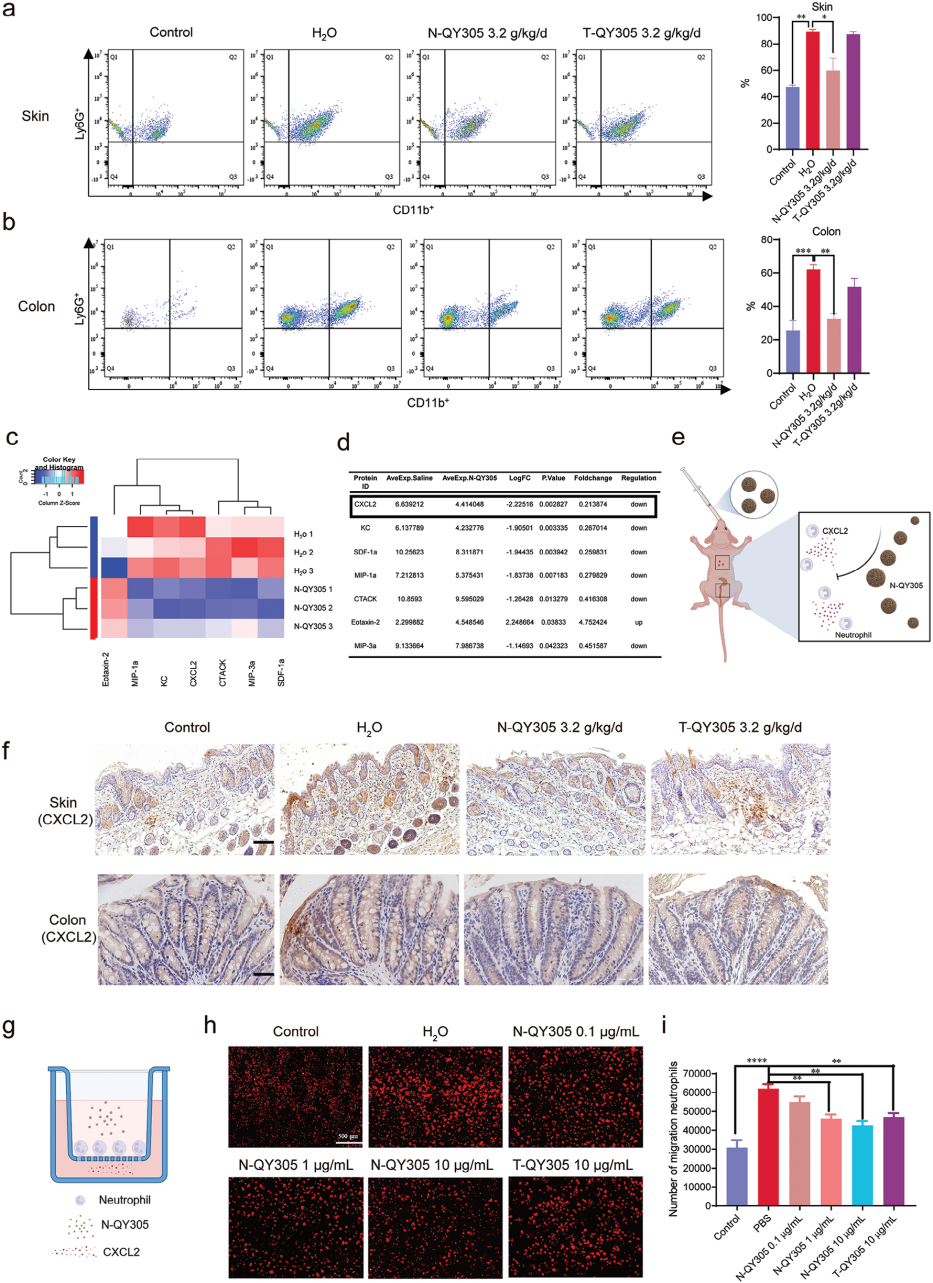
N‐QY305 reduced the adverse reaction by regulating neutrophil migration. a,b) Flow cytometry for neutrophil in skin and colon tissue from control, H_2_O, N‐QY305 3.2 g kg ^−1^d^−1^ and T‐QY305 3.2 g kg ^−1^d^−1^ group. The fluorescence distribution of CD11b^+^ and Ly6G^+^ in CD45^+^ gated cells and neutrophil was defined as CD45^+^CD11b^+^Ly6G^+^. The distribution of cells as a percentage of CD45^+^cells was shown in each quadrant, as well as the statistical data analysis (n≥3). c,d) Skin protein levels of H_2_O and N‐QY305 group were measured by RayBiotech Mouse Chemokine Array Q1 (n = 3). RayBiotech Mouse 25 Chemokines Array differential protein expression results were shown by Cluster heatmap and a table with a high to low significance level. e) Schematic illustration of the mechanism of N‐QY305. f) Skin tissue representative immunohistochemistry images of CXCL2 (scale bar = 100 µm, top row, n = 3) and colon tissue representative immunohistochemistry images of CXCL2 (scale bar = 50 µm, lower row, n = 3). g) Schematic of the Transwell migration assay. Cells were seeded in the upper chamber while CXCL2 was added in the lower chamber. N‐QY305 and T‐QY305 were added in the upper chamber. h) Fluorescent images of neutrophil (DiD‐labeled) in the lower chambers with and without N‐QY305 or T‐QY305. i) The number of neutrophil migrations. Data are presented as means ± SEM. ^*^ *p* <0.05, ^**^ *p* <0.01, “^*^” compared with model.

Thus, next we explore how N‐QY305 can play the key role in the treatment of cutaneous adverse reaction and diarrhea better than T‐QY305. For this purpose, we compared the proportions of neutrophil recruitment in inflamed skin and colon tissue, using the same dose of N‐QY305 and T‐QY305. Similar to the results obtained regarding its efficacy, N‐QY305 reduced neutrophil recruitment in inflamed tissue of skin and colon with a higher efficacy than T‐QY305 (**Figure** **6**a,b). Next, we analyzed neutrophil recruitment from a deeper biochemical perspective, by analyzing the interaction between migrating leukocytes and chemokines located on the surface of the mice skin. Due to the short course and less incidence rate of diarrhea than cutaneous adverse reaction in clinical, we detected 25 chemokines only in skin tissue. The results showed that the top two differential chemokines were neutrophil‐specific (Figure 6c,d), among which CXCL2 not only has a chemotactic effect on neutrophil, but can also be released by neutrophil.^[^ ^25^ ^]^ Therefore, to confirm the correlation between the abundance of CXCL2 surface chemokines and the proclivity of the mice skin to be infiltrated by neutrophil, we proceeded to the immunohistochemistry and Transwell experiments. The results demonstrated that N‐QY305 reduced the expression of CXCL2 in skin and colon tissue, which showed the same trend as flow cytometry results (Figure 6f). Consequently, the Transwell assay confirmed the efficacy of N‐QY305 in reducing the recruitment of neutrophil to CXCL2 in vitro and showed more effectiveness than T‐QY305 at the same dose (Figure 6g–I). These data suggested that N‐QY305 is much more potent than T‐QY305 upon the inhibition of CXCL2 expression in skin and colon tissue, and eventually presents better features to prevent skin and colon tissue for being infiltrated by neutrophil (Figure 6e).

In addition, N‐QY305 could promote the proliferation of the keratinocytes and colon epithelial cells in vitro (Figure S17, Supporting Information), thus confirming its involvement in the restoration mechanism of skin and colon tissue from the damage caused by the treatment. In regard to the obtained results, we were able to conclude that the treatment of cutaneous adverse reaction and diarrhea through N‐QY305, may lead to positive outcomes that are the significant inhibition of neutrophil infiltration in skin and colon tissue, along with the restoration of their damaged tissue.

## 3 Conclusion

The carried study proposed a new therapeutic strategy based on the TCM, for the alleviation of cutaneous adverse reaction and diarrhea induced by EGFRIs‐related treatment. In the particular case of our study, we aimed to assess the pharmacodynamic basis of T‐QY305, and clarified the significance of nanostructures in Chinese medicine formula, according to the importance of their function in the mechanism of inhibition of cutaneous adverse reaction and diarrhea induced by EGFRIs. The study revealed that the pharmacodynamic feature of T‐QY305 originates from one of its subunits labeled as N‐QY305 nanostructure. Indeed, further analysis demonstrated the high potency of N‐QY305 to inhibit the main cause of adverse reactions, namely regulation of CXCL2 expression in skin and colon tissue, and eventually presenting better features to prevent skin and colon tissue for being infiltrated by neutrophil. Regarding these capital functions, mediated by N‐QY305, we were able to hypothesize that, N‐QY305 represented the “*King*” herb (“*Jun*”) of the “*Jun*”‐“*Chen*”‐“*Zuo*”‐“*Shi*” theory in our case; the decoction helping for the self‐assembling of the nanostructure can be considered as the minister (“*Chen*”); meanwhile the boiling process mediating the completion of the nanostructure formation may be playing the role of the assistant (“*Zuo*”); and the rest of the compounds, although not fully known can be regarded as the conveyer (“*Shi*”). Thus, from our study, TCM research may further be considered as significantly related to nanomedicine, paving the way for further studies based on nanotechnology.

However, it is to be mentioned that our study presents certain limitations, such as the non‐consideration of other inflammatory cells upon N‐QY305 inhibitory mechanism of cutaneous adverse reaction and diarrhea. Indeed, our work represents a gateway for other groups of research, which may aim to approach the issue of adverse reactions from another angle, such as the analysis of the impact of macrophages in the regulation of skin and colon inflammation induced by EGFRIs.^[^ ^18^, ^26^ ^]^ Doing so, the availability of data related to the treatment of cutaneous adverse reaction and diarrhea induced by EGFRIs therapy, may be enhanced and promote the developing field of TCM research.

## 4 Experimental Section

### Materials

T‐QY305 (Beijing Tcmages Pharmaceutical Co., Ltd), gefitinib (IRESSA, AstraZeneca), minocycline (Minocin, Hanhui Pharmaceuticals Co., Ltd), loperamide (Imodium, Xian Janssen pharmaceutical Ltd)

### Preparation and Characterization of N‐QY305

*Astragalus membranaceus* 30 g, *Lonicera japonica* 30 g, *Angelica sinensis* 30 g, *Licorice* 10 g, and *centipede* 1 g, were soaked in cold water for 30 min and boiling for 60 min and then the extracts were filtered through gauze. For gradient centrifugation, the precipitate of decoction was discarded, and the supernatant was centrifuged again after centrifugation for 30 min at a centrifugal speed of 4000/6000/8000/10 000 rpm. Then the supernatant was dialyzed against deionized water (MWCO = 3.5 kDa). The morphology was measured by transmission electron microscopy (TEM, Hitachi‐HT7700) and scanning electron microscope (SEM, FEI‐NOVA Nano SEM 430 +EDS), size distribution and zeta potential were measured by dynamic light scattering (Malvern zeta sizer, Malvern). Identification of N‐QY305 was detected by LC‐MS/MS.

### Therapeutic Efficacy of T‐QY305/N‐QY305 on Cutaneous Adverse Reaction and Diarrhea in balb/c Nude Mice Carrying Tumor

The cutaneous adverse reaction and diarrhea model of male balb/c nude mice (19–20 g) was created as in the previous study.^[^ ^10^, ^27^ ^]^ First, an orthotopic non‐small cell lung cancer model was used, which was described in Supplementary Material. All balb/c nude mice carrying tumor randomly assigned to six groups and administered the following formulations: control group (nontreatment), H_2_O group (gefitinib 200 mg kg^−1^ d^−1^), T‐QY305‐L group (gefitinib+T‐QY305 4 g kg^−1^ d^−1^), T‐QY305‐H group (gefitinib+T‐QY305 8 g kg^−1^ d^−1^), minocycline group (gefitinib+ minocycline 50 mg kg^−1^ d^−1^), loperamide group (gefitinib+ loperamide 6.4 mg kg^−1^ d^−1^). All drugs were dissolved in H_2_O and orally administered daily, over a 4 h dosing interval. Diarrhea scores were measured and recorded every day. On day 8, the animals’ facial and dorsal skin were photographed, and the skin moisture was detected before being euthanized. Then, after euthanized, the skin and colon tissue of each mouse were fixed, sectioned, and stained with H&E or immunofluorescence, followed by super‐resolution images using Leica Aperio VERSA 8 digital pathology scanner (Leica Biosystems, Buffalo Grove) and analyzed by Aperio ImageScope (v12.3.2.5030, Leica Biosystems).

For the observation of N‐QY305 effects, balb/c nude mice carrying tumor randomly assigned to five groups, control group (nontreatment), H_2_O group (gefitinib 200 mg kg^−1^ d^−1^), N‐QY305‐L group (gefitinib+N‐QY305 1.6 g kg^−1^ d^−1^), N‐QY305‐H group (gefitinib+N‐QY305 3.2 g kg^−1^ d^−1^), T‐QY305 group (gefitinib+T‐QY305 3.2 g kg^−1^ d^−1^). All drugs were dissolved in H_2_O and orally administered daily, over a 4 h dosing interval. Body weight was measured and recorded every 2 days. On day 10, animals were euthanized, facial and dorsal skin were photographed, and skin moisture was detected before being euthanized. Then, after euthanized, the skin and colon tissue of each mouse were analyzed by flow cytometry, stored at −80 °C for protein‐chip technique or fixed, sectioned, and stained with H&E or immunohistochemistry.

### Therapeutic Efficacy of T‐QY305 on Cutaneous Adverse Reaction and Diarrhea in BN Rats

The cutaneous adverse reaction and diarrhea model of female Brown Norway (BN) rats (150–200 g) was created as in the previous study.^[^ ^18^ ^]^ Briefly, rats were divided into five groups, control group (nontreatment), H_2_O group (gefitinib 200 mg kg^−1^ d^−1^), T‐QY305‐L group (gefitinib+T‐QY305 4 g kg^−1^ d^−1^), T‐QY305‐H group (gefitinib+T‐QY305 8 g kg^−1^ d^−1^). All drugs were dissolved in H_2_O and orally administered daily, over a 4 h dosing interval. Body weight was measured and recorded every 3 days. On day 33, animals were euthanized, and rats’ facial and dorsal skin were photographed before being euthanized. The diarrhea score was measured on days 7 and 28, respectively. Then, after euthanized, the skin and colon tissue of each rat were fixed, sectioned, and stained with H&E or immunohistochemistry.

### Skin Moisture Measurement

For deep skin moisture measurement of balb/c nude mice, Delfin MoistureMeterEpid (Delfin Technologies, Kuopio) was used. Skin moisture measurement was assessed on the facial and dorsal skin. The probe was removed from the skin and replaced, after which a second measurement was taken. An average of the two readings was used as the skin moisture for each mouse.

### Immunohistochemistry and Immunofluorescence Experiments

Immunohistochemistry experiments were performed following standard procedures. Briefly, paraffin sections of 5 µm were baked at 60 °C for 2 h, deparaffinized, hydrated using decreasing ethanol series and subjected to microwave heat‐induced antigen retrieval using sodium citrate buffer pH   6.0. Sections were then washed with PBS (3 × 3 min) and endogenous peroxidase activity was blocked by peroxidase block for 10 min. Next, sections were washed with PBS (3 × 3 min) and blocked in 5 % normal goat serum in PBS for 60 min at room temperature, followed by incubation with primary antibodies for 1 h at 37 °C. Then sections were washed with PBS (3 × 3 min) and incubated with reaction enhancer solution and HRP‐labeled secondary antibodies (goat anti‐rabbit/mouse IgG) at room temperature for 20 min, respectively. After washed with PBS (3 × 3 min), color reaction of sections was developed for 5 min using DAB. Then it was followed by nuclear counterstaining with Mayer's hematoxylin, dehydrated and transparent. The images were captured by Leica Aperio VERSA 8 digital pathology scanner and analyzed by Aperio ImageScope. The primary antibodies used were: anti‐TNF‐α (NB600‐587, Novus Biologicals, 1:500), anti‐myeloperoxidase (ab208670, Abcam, 1:500), anti‐IL‐8 (27095‐1‐AP, Proteintech, 1:200), CXCL2 (701 126, Invitrogen,1:100).

For immunofluorescence experiments, the same procedure as immunohistochemistry assay before microwave heat‐induced antigen retrieval. Sections were then washed with PBS (3 × 3 min) and covered the objective area with goat serum working solution to block non‐specific binding for 60 min. Throw away the blocking solution slightly and incubate slides with primary antibody overnight at 4 °C. Next, sections were washed with PBS (3 × 3 min) and incubated with secondary antibody for 60 min at room temperature in dark condition. Then sections were washed with PBS (3 × 3 min) and incubated with DAPI solution at room temperature for 10 min in dark condition. After washed with PBS (3 × 3 min), slides were mounted with anti‐fade mounting medium. Anti‐myeloperoxidase mouse mAb (GB12224, Servicebio, 1:100), anti‐Ly6G Rabbit pAb (GB11229, Servicebio, 1:100), Cy3 conjugated Goat Anti‐mouse IgG (H+L) (GB21301, Servicebio, 1:200), FITC IgG (H+L) (FITC conjugated Goat Anti‐Rabbit IgG (H+L) (GB22303, Servicebio, 1:200).

### Flow Cytometry Analysis

To test the percentage of neutrophil recruitment in skin and colon tissue from mice, flow cytometry acquisition was performed with the FACSVerse (BD Biosciences) and the stained cells were analyzed with flow cytometry software. First, single‐cell suspensions were obtained from skin and colon of balb/c nude mice, and incubated with mouse TruStain fcX Antibodies (Cat#101 319, Biolegend) for 10 min at 4 °C prior to immunostaining. Then cells were stained with fluorescently labeled antibodies with 1:100 dilutions including PerCP/Cyanine5.5 anti‐mouse Ly6G Antibody (Cat#127 615, BioLegend), APC anti‐mouse/human CD11b Antibody (Cat#101 211, BioLegend) and PE anti‐mouse CD45 Antibody (Cat#103 105, BioLegend).

### Quantitative Measurement of 25 Mouse Chemokines

Mice skin from H_2_O and N‐QY305 group were frozen at −80 °C until use. Skin was digested in a cell lysis buffer containing a protease inhibitor cocktail and the protein concentration was determined by BCA method (BCA Protein Assay Kit, Pierce Thermo Scientific, 23227). The supernatant was collected for chemokine array detection using Quantibody Mouse Chemokine Array 1 (Cat# QAM‐CHE‐1, RayBiotech, Inc.) following the manufacturer's instructions. Sample diluents were added into each well to block slides for 1 h, decant buffer from each well and add 100 µL standard chemokines or samples to each well. Incubate arrays at 4 °C overnight. After washing, the array was incubated with 80 µL detection antibody cocktail at room temperature for 2 h. Washing again, 80 µL of Cy3 equivalent dye‐conjugated streptavidin was added to each well at room temperature for 2 h in the dark. Wash and collect the fluorescence intensities by InnoScan 300 Microarray Scanner and the data quantitative analysis was conducted using microarray analysis software (GenePix, ScanArray Express, ArrayVision, MicroVigene, etc.).

### Transwell

The migration ability of neutrophil to CXCL2 was measured using a Transwell with 3 µm‐pore polyester membrane inserts, with 2 × 10^5^ DiD‐labeled neutrophil seeding in the upper chamber, and 10 ng CXCL2 (20 ng mL^−1^) added to the lower chamber. After 1 h, fluorescent images of the lower chamber were taken using an inverted fluorescence microscope (Leica, DMi8‐M).

### Animal Ethics Statement

The experiments described were carried out at Laboratory Animal Center of Beijing University of Chinese Medicine. All animal experiments were approved by the Ethics Committee of Beijing University of Chinese Medicine (approval reference number is BUCM‐4‐2020101102‐4185, BUCM‐4‐2021101104‐4141). All procedures were performed under inhalation anesthesia using isoflurane. All efforts were made to minimize animal suffering and to reduce the number of animals used. Each animal was given unlimited food and water throughout all experiments.

### Statistical Analysis

The data were presented as means ± standard error (SEM). Analysis was processed by GraphPad prism 8.0. Statistical significance was determined by ordinary one‐way ANOVA test and two‐way ANOVA test with multiple comparisons (Dunn's post‐test). P values less than 0.05 was considered statistically significant.

## Conflict of Interest

The authors declare no conflict of interest.

## Supporting information

Supporting Information

## Data Availability

The data that support the findings of this study are available in the supplementary material of this article.
