# Supplementary material for: Traditional Chinese Medicine Formulae QY305 Reducing Cutaneous Adverse Reaction and Diarrhea by its Nanostructure
Source: Adv Sci (Weinh). 2023 Dec 3;11(5):2306140. doi: 10.1002/advs.202306140 (PMC10837375; doi:10.1002/advs.202306140)
Supplement: Supplementary file 1 — Supporting Information [file ADVS-11-2306140-s001.pdf]

ADVANCED SCIENCE
Open Access

Supporting Information

for *Adv. Sci.*, DOI 10.1002/advs.202306140

Traditional Chinese Medicine Formulae QY305 Reducing Cutaneous Adverse Reaction and Diarrhea by its Nanostructure

Ya-Li Zhang, Ya-Lei Wang, Ke Yan, Haiyan Li, Xinyu Zhang, Julien Milon Essola, Chengcheng Ding, Kexin Chang, Guangchao Qing, Fuxue Zhang, Yan Tan, Tiantian Peng, Xu Wang, Miao Jiang, Xing-Jie Liang and Qian Hua**

Supplementary Information

Traditional Chinese Medicine Formulae QY305 Reducing Cutaneous Adverse Reaction and Diarrhea by Its Nanostructure

Ya-Li Zhang † [1, 2], Ya-Lei Wang † [1], Ke Yan [1], Haiyan Li [1], Xinyu Zhang [1], Julien Milon Essola [2], Chengcheng Ding [1], Kexin Chang [1], Guangchao Qing [2], Fuxue Zhang [2], Yan Tan [1], Tiantian Peng [1], Xu Wang [1], Miao Jiang [1], Xing-Jie Liang [2], Qian Hua* [1]*

[1] School of Life Sciences, School of Traditional Chinese Medicine, Beijing University of Chinese Medicine, Beijing, 102488, China.

[2] CAS Center for Excellence in Nanoscience, CAS Key Laboratory for Biomedical Effects of Nanomaterials and Nanosafety, Chinese Academy of Sciences and National Center for Nanoscience and Technology of China, Beijing, 100190, China.

†/* These authors contributed equally to the manuscript.

* Address correspondence to:

Xing-Jie Liang, Ph.D., Professor, CAS Center for Excellence in Nanoscience, CAS Key Laboratory for Biomedical Effects of Nanomaterials and Nanosafety, Chinese Academy of Sciences and National Center for Nanoscience and Technology of China, Beijing, 100190, China, E-mail: liangxj@nanoctr.cn;

Qian Hua, Ph.D., Professor, School of Life Sciences, Beijing University of Chinese Medicine, Beijing, 102488, China, E-mail: huaq@bucm.edu.cn.

This PDF file includes:

— Supplementary Figure 1-17

Other supplementary materials for this manuscript include the following:

— Supplementary Table 1-5

Results

Development of an adverse reaction model in balb/c nude mice. Balb/c nude mice carrying tumor was divided into five groups, control (n=4), model (n=6), gefitinib 100 mg/kg (n=6), gefitinib 200 mg/kg (n=6) and gefitinib 300 mg/kg (n=6) groups (Figure S2a). After confirming the successful establishment of the tumor model on day 7 (0 day of treatment) by *in vivo* bioluminescence imaging (Figure S2b), mice were treated orally administered daily with H_2O (Model group), gefitinib 100 mg/kg, 200 mg/kg or 300 mg/kg. The antitumor efficacy was evaluated and gefitinib exhibited an effect on tumor relative growth rate (fluorescence intensity of 14 d/7 d) compared with model group (Figure S2c).

Further, we observed the skin phenotype induced by various concentrations of gefitinib on day 7 (Figure S3), as compared to model group, gefitinib at 200 mg/kg and 300 mg/kg occurring cutaneous adverse reaction (Figure S3a, b) and obviously increased TEWL in dorsal, neck and facial (Figure S3c).On day 14 (Figure S4), as compared to model group, gefitinib at 300 mg/kg occurred cutaneous adverse reaction (Figure S4a, b) and obviously increased TEWL in dorsal, neck and facial; gefitinib at 200 mg/kg also occurred obvious cutaneous adverse reaction and only obviously increased TEWL in facial (Figure S4c). For diarrhea, the time to first occurrence (any grade) was day 4 after gefitinib treatment, and compared with the model group, gefitinib-treated groups' mean diarrhea scores were higher (Figure S5a), which also showed upper colon bleeding (Figure S5b). Combined with all above experimental results of occurrence and severity of cutaneous adverse reaction and diarrhea, we chose gefitinib at 200 mg/kg as the dose modeling in balb/c nude mice carried tumor. It should be illustrated that two mice of model group and three of gefitinib 100 mg/kg group died under anesthesia.

Experimental Section

Clinical retrospective study. This study was an investigator-initiated, single-site, retrospective analysis clinical study. Patients with cutaneous adverse reaction induced by EGFRIs therapy, visiting Dongzhimen Hospital Affiliated to Beijing University of Chinese Medicine between 1 Oct 2021 and 30 Oct 2022 were involved without any further selection. We assessed the degree of cutaneous adverse reaction according to the National Cancer Institute Common Terminology Criteria for Adverse Events (CTCAE) version 5.0, 0: no rash/drying/pruritus; 1: mild rash/drying/pruritus; 2: moderate rash/drying/pruritus; 3: severe rash/drying/pruritus.

clinical characteristics of two patients.

Patient 1: The baseline characteristics and clinical features of patient 1 were shown in Table S1 and Fig.1 B. A 66-year-old male was diagnosed with hepatic metastasis with EGFR mutation of colonic moderately-differentiated adenocarcinoma in 2019, and he began his treatment in 2021 by intravenous administration of cetuximab (an EGFR monoclonal antibody). Within the starting period of cetuximab therapy, the patient developed an acneiform reaction on his back. And his skin score was evaluated on the first visit in September 2022 (rash: grade 3, drying: grade 2, pruritus: grade 3). Then he received T-QY305 orally administered in two divided doses, on a daily basis for 14 days, with significant symptomatic improvement (rash: grade 2, drying: grade 1, pruritus: grade 2).

Patient 2: The baseline characteristics and clinical features of patient 2 were shown in

Table S2 and Fig. S1 A. A 60-year-old female was diagnosed with lung adenocarcinoma in 2015 and she began her treatment on September 2021, by a once-a-day daily administration of 40 mg of second-line afatinib (an irreversible EGFR-TKI). Within less than one month of the starting period of afatinib therapy, the patient developed an acneiform reaction on her forehead and the skin score was evaluated on the first visit in October 2021 (rash: grade 1, drying: grade 1, pruritus: grade 2). Then she received T-QY305 orally daily in two divided doses for 14 days, with significant pruritus symptomatic improvement (rash: grade 1, drying: grade 1, pruritus: grade 1). And her forehead rash improved only with melanin pigmentation at a follow-up visit in December 2021.

Generation of orthotopic non-small cell lung cancer model mouse model. For *in vivo* tumor growth generation (a basis of adverse reaction model), tail vein injection of PC9-luc cells (1×10^5) was performed in blindly randomized male balb/c nude mice (18-20 g, n≥4 per group). After 7 days and before the mice being euthanized, optical imaging was performed with an IVIS Spectrum small-animal *in vivo* imaging system (Caliper LifeSciences, Hopkinton) with integrated isoflurane anesthesia.

Cell viability assay. Cell viability assay was conducted using Cell Counting Kit (CCK-8) (LABLEAD) following the manufacturer's instructions. HaCaT cells were cultured in 96-well plates (3000 cells/well) and incubated in the absence or presence of various concentrations of gefitinib (0, 0.016, 0.08, 0.4, 2, 10, 50 μM) and/or N-QY305 (0.0001μg/mL, 0.1 μg/mL). NCM460 cells were cultured in 96-well plates (3000 cells/well) and incubated in the absence or presence of various concentrations of gefitinib (0, 10, 20, 30, 40, 50 μM) and/or N-QY305 (10 μg/mL, 100 μg/mL).

LC-MS/MS. Analysis of standards, T-QY305 and N-QY305 were performed on a triple quadrupole tandem liquid chromatography-mass spectrometry (LC-MS/MS) system (LCMS8050, Shimadzu Corporation). The T-QY305 (25 mg dissolved in 1 mL pure water) and N-QY305 freeze-dried powder (2.5 mg dissolved in 1 mL pure water) were collected by ultrasonic extraction for 30 min and then passed through a 0.22 μm filter membrane for LC-MS/MS analysis. The separation was performed on a Waters ACQUITY UPLC BEH C18 (2.1 mm×100 mm, 1.7 μm) column and elution was performed with mobile phases of 0.1% formic acid (aqueous phase A) and acetonitrile (organic phase B) in gradient mode. The proportion of acetonitrile varied from 10 to 25% in 20 min (0-10 min, 90-75% A, 10-25% B; 10-20 min, 75-30% A, 25-70% B) at a flow rate of 0.5 mL/min; each injection volume was set to 1 μL. Mass spectrometry conditions and parameters: electrospray ionization (ESI) source: negative ionization mode (m/z 100-1500), the ion source temperature (TEM): 500°C, ion spray voltage (IS): 4500 V, ion source gas 1 (GS1): 60 psi, ion source gas 2 (GS2): 60 psi.

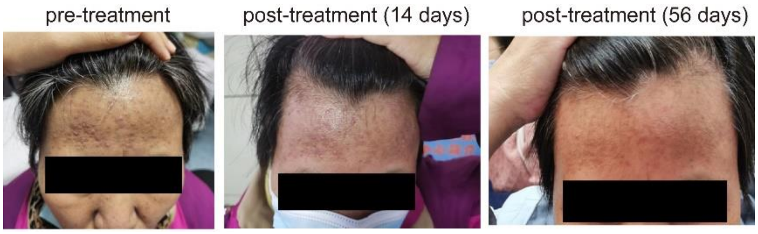

Figure S1. T-QY305 reduced the cutaneous adverse reaction induced by afatinib in cancer patient 2. Clinical features of patient 2: pre-treatment and post-treatment with a daily oral administration of T-QY305 for 14 days.

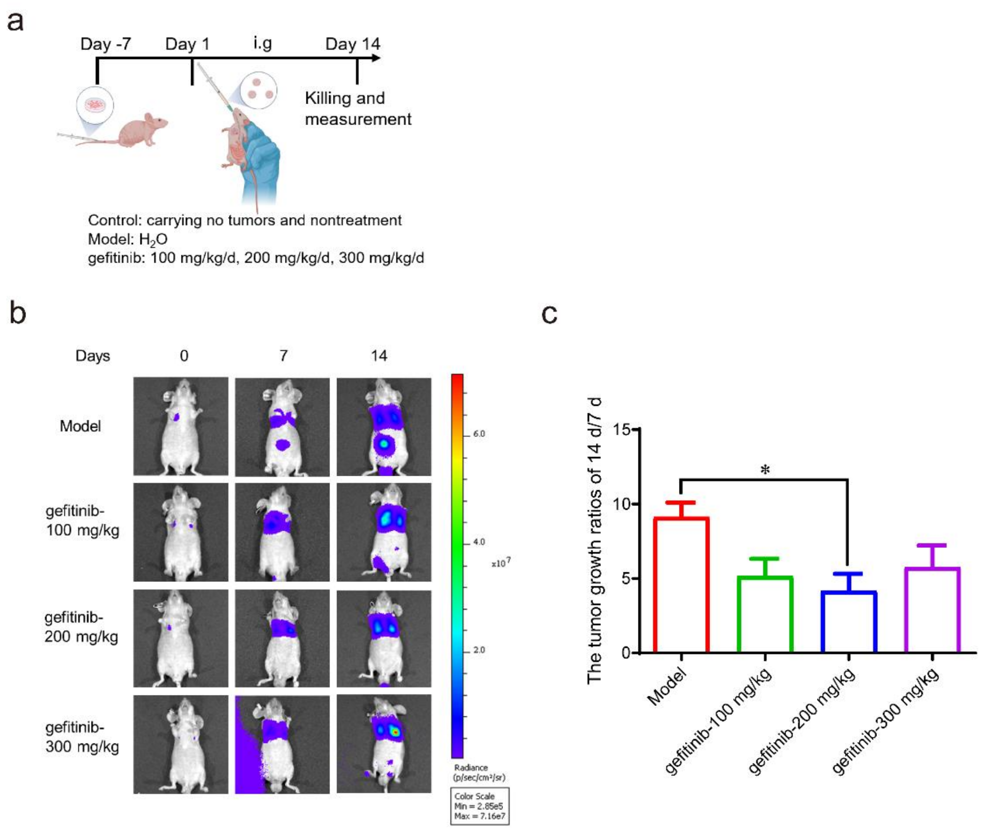

Figure S2. Gefitinib showed antitumor effect in balb/c nude mice. **a** Experimental timeline of cutaneous adverse reaction and diarrhea induced by gefitinib in balb/c nude mice carrying tumor. Balb/c nude mice with no treatment as control group, tail vein injected with PC9-Luc cells and administrated with H_2O as model group, tail vein injected with PC9-Luc cells and administrated with gefitinib 100, 200, 300 mg/kg/d as gefitinib groups, followed by a daily oral administration for 14 days. **b** Bioluminescent imaging of orthotopic lung metastasis mouse model after treatment with gefitinib (100 mg/kg, 200 mg/kg, 300 mg/kg) on days 0, 7 and 14. **c** The tumor growth was assessed by the fluorescence intensity ratio of day 14/7 (n≥3). Data are presented as means ± SEM. *$p<0.05$, "*" compared with H_2O group.

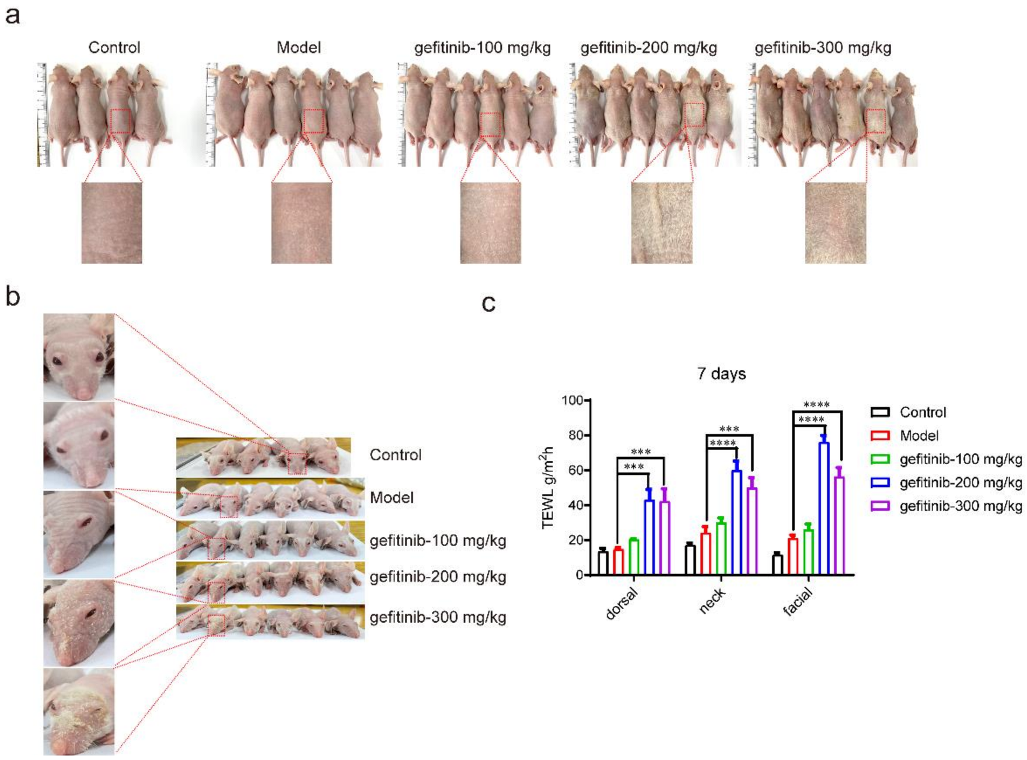

Figure S3. Gefitinib induced cutaneous adverse reaction in balb/c nude mice carrying tumor on day 7. **a, b** The phenotype of dorsal and facial skin was captured by mobile devices on day 7 (n≥4). **c** Transepidermal water loss (TEWL) assessed by Tewameter TM Hex on day 7 (n≥4). Data are presented as means ± SEM. ***p<0.005, ****p<0.001, "*" compared with H_2O group.

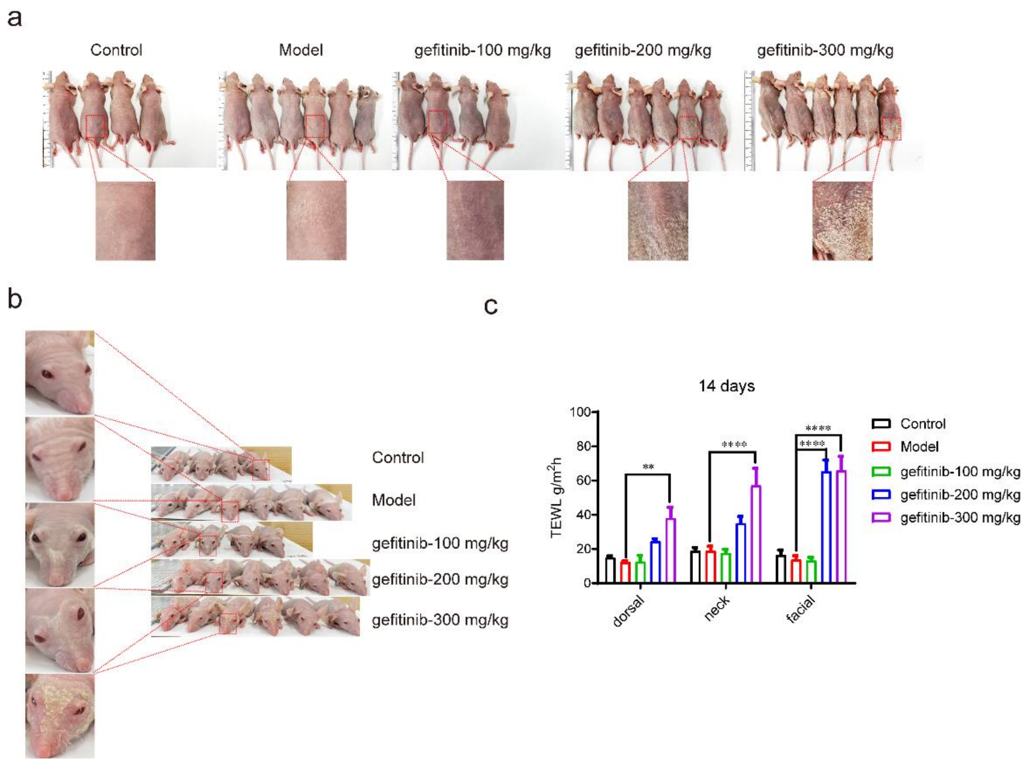

Figure S4. Gefitinib induced cutaneous adverse reaction in balb/c nude mice carrying tumor on day 14. **a, b** The phenotype of dorsal and facial skin was captured by mobile devices on day 14 (n≥4). **c** TEWL assessed by Tewameter TM Hex on day 14 (n≥3). Data are presented as means ± SEM. **p<0.01, ***p<0.005, ****p<0.001, “*” compared with H_2O group.

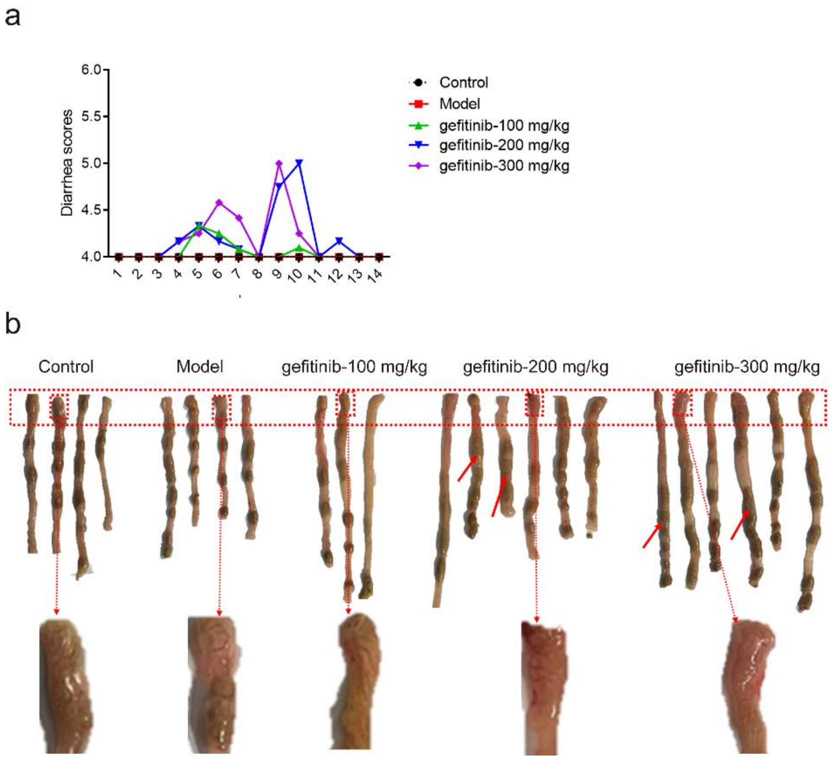

Figure S5. Gefitinib induced diarrhea in balb/c nude mice carrying tumor. **a** The severity of diarrhea was scored daily followed by Bristol Stool Scale (n≥3): score 4, normal; score 5, mild diarrhea; score 6, moderate diarrhea; score 7, severe diarrhea. **b** The phenotype of colon was captured by mobile devices and the zoom-in regions showing the upper colon (Red array indicates the shapeless stool) (n≥3).

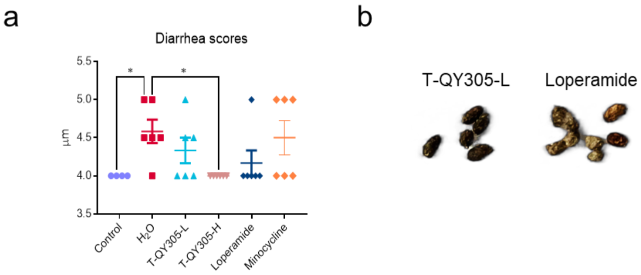

Figure S6. Minocycline and loperamide showed no better results than T-QY305 on the whole. **a** The severity of diarrhea (on day 5) was scored followed by Bristol Stool Scale (n≥4): score 4, normal; score 5, mild diarrhea; score 6, moderate diarrhea; score 7, severe diarrhea. **b** The different phenotype of mouse stool between T-QY305 group and loperamide group. The stool of loperamide group presented higher level of dryness and hardness phenotype than those of the T-QY305 group.

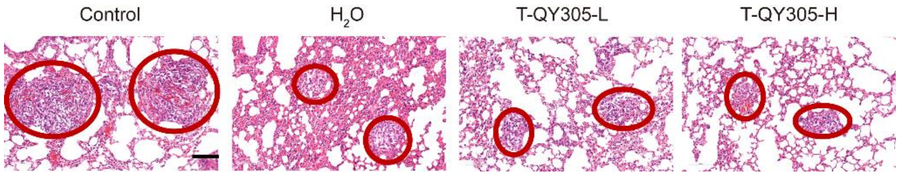

Figure S7. T-QY305 had no negative impact on gefitinib antitumor effect in balb/c nude mice. Lung tumor nodes were observed by H&E staining and the red circle showed the tumor lesions, scale bar=100 μm.

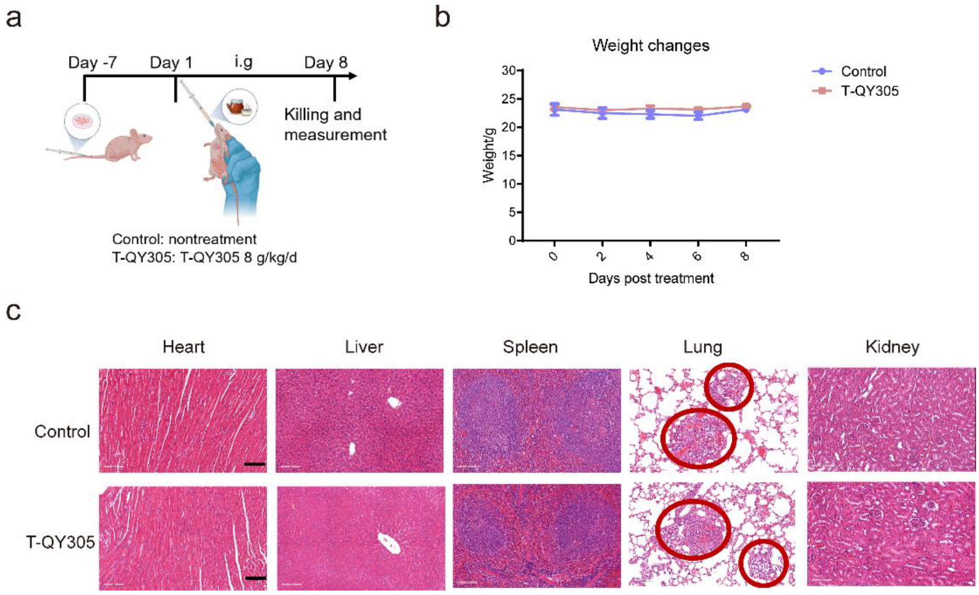

Figure S8. T-QY305 had no toxicity on major organs in balb/c nude mice. **a** Experimental timeline for T-QY305 treatment on balb/c nude mice carrying tumor. **b** The body weight was measured and recorded every 2 days (n=4). **c** H&E staining of histological sections from major organs, including heart, liver, spleen, lung and kidney (n=3), scale bar=100 μm.

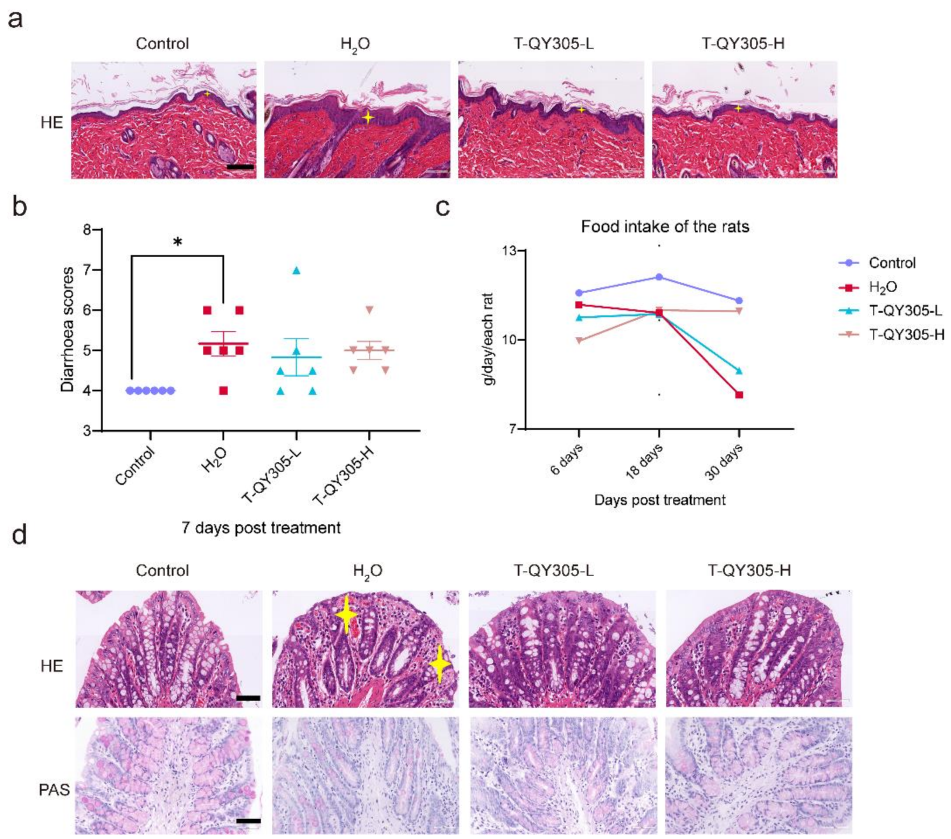

Figure S9. T-QY305 reduced the phenotype of skin and colon tissue in BN rats. **a** The thickness of the skin epidermis was detected by H&E staining (n=3), scale bar=100 μm. **b** The severity of diarrhea was scored on day 7 by Bristol Stool Scale (n=6): score 4, normal; score 5, mild diarrhea; score 6, moderate diarrhea; score 7, severe diarrhea. **c** The food intake was measured on days 6, 18, 30. **d** Top row: the phenotype of colon tissue detected by H&E staining (n=3), the stars above colon represented the damaged area, scale bar=50 μm. Lower row: PAS (Periodic Acid Schiff) stained of goblet cells in colon tissue for each group (n=3), scale bar=50 μm. Data are presented as means ± SEM, *$p<0.05$, "*" compared with H_2O group.

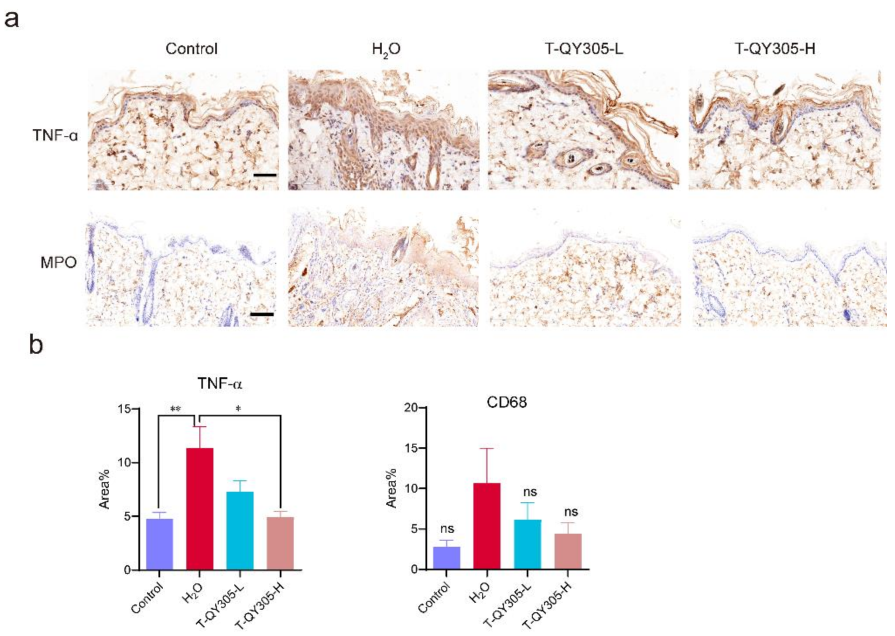

Figure S10. T-QY305 reduced the infiltration of TNF-α and the recruitment of macrophages in skin tissue of BN rats. **a** Top row: Skin tissue representative immunohistochemistry images of inflammatory cytokine TNF-α, n=3, scale bar=50 μm. Lower row: Skin tissue representative immunohistochemistry images of the marker of macrophages ($CD68^+$), scale bar=100 μm. **b** Skin tissue immunohistochemistry results are presented as means ± SEM, n=3. *p<0.05, **p<0.01, "*" compared with H_2O group.

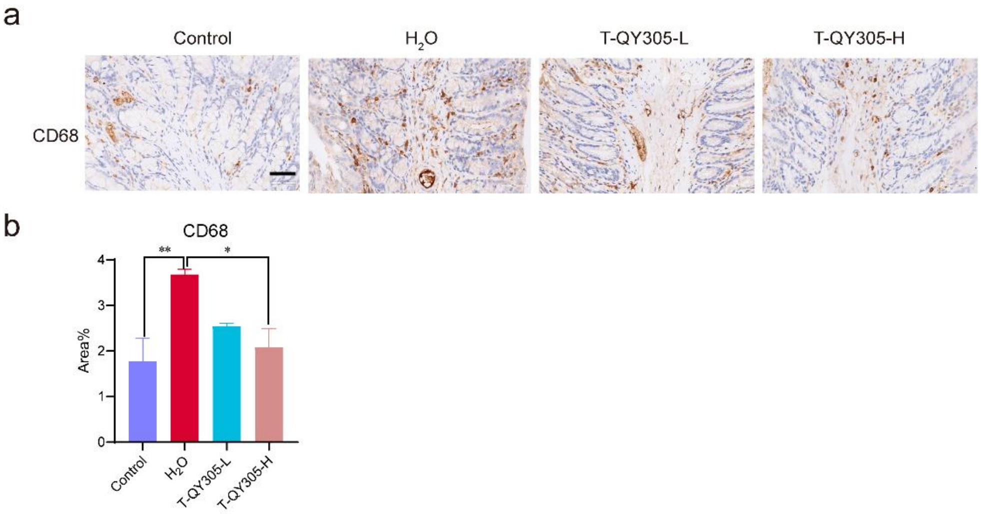

Figure S11. T-QY305 reduced the recruitment of macrophages in colon tissue of BN rats. **a** Colon tissue representative immunohistochemistry images of the marker of macrophages ($CD68^+$), scale bar=50 μm. **b** Colon tissue immunohistochemistry results are presented as means ± SEM, n=3. *p<0.05, "*" compared with H_2O group.

a

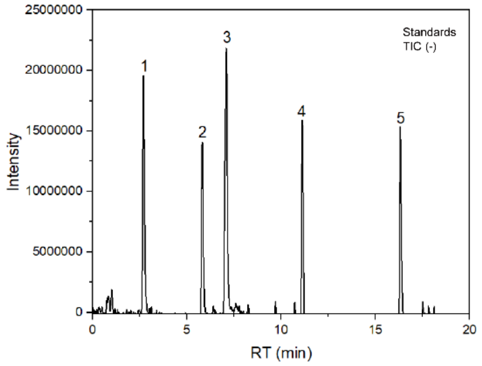

b

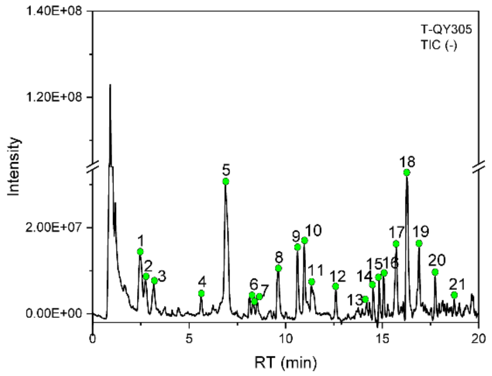

Figure S12. Liquid Chromatograph-Mass Spectrometry (LC-MS/MS) of standards and T-QY305. **a** Analyses of 5 standards by LC-MS/MS and also listed in Table S3. **b** Analyses of 21 compounds from T-QY305 by LC-MS/MS and also listed in Table S4.

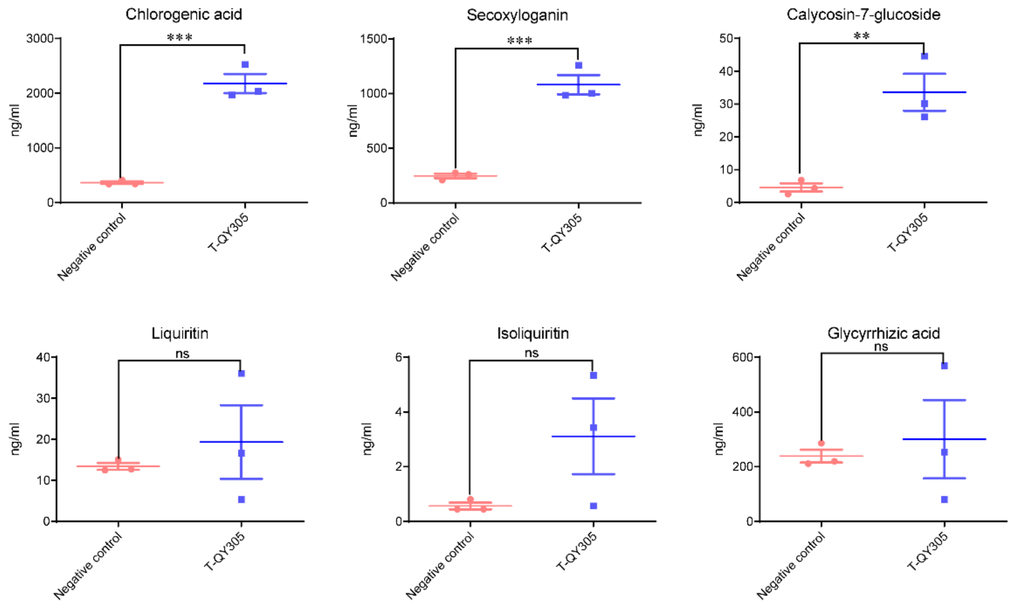

Figure S13. Compounds of T-QY305 distributed more to the inflammation sites. Negative control: Balb/c nude mice were orally administered daily with T-QY305 for 8 days, T-QY305 group: Balb/c nude mice were orally administered daily with gefitinib and T-QY305 for 8 days. Skin tissue was collected to compare the content difference (chlorogenic acid, secoxyloganin, calycosin-7-glucoside, liquiritin, isoliquiritin, glycyrrhizic acid) between negative control and T-QY305 group by LC-MS/MS.

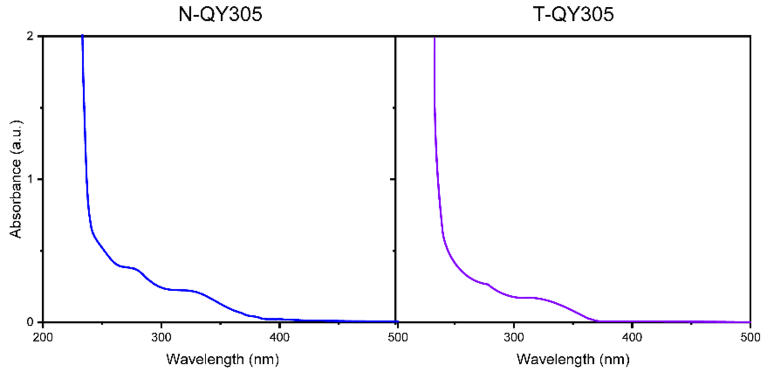

Figure S14. N-QY305 has a similar stability to T-QY305 in SGF. N-QY305 and T-QY305 were incubated in SGF for 2 h at 37°C with shaking, and then samples were collected to test UV-visible spectra after filtered through 0.22 μm microporous membrane.

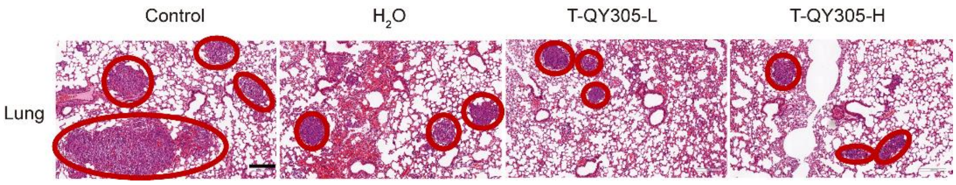

Figure S15. N-QY305 had no negative impact on gefitinib antitumor effect in balb/c nude mice. Lung tumor nodes were observed by H&E staining and the red circle showed the tumor lesions, scale bar=100 μm.

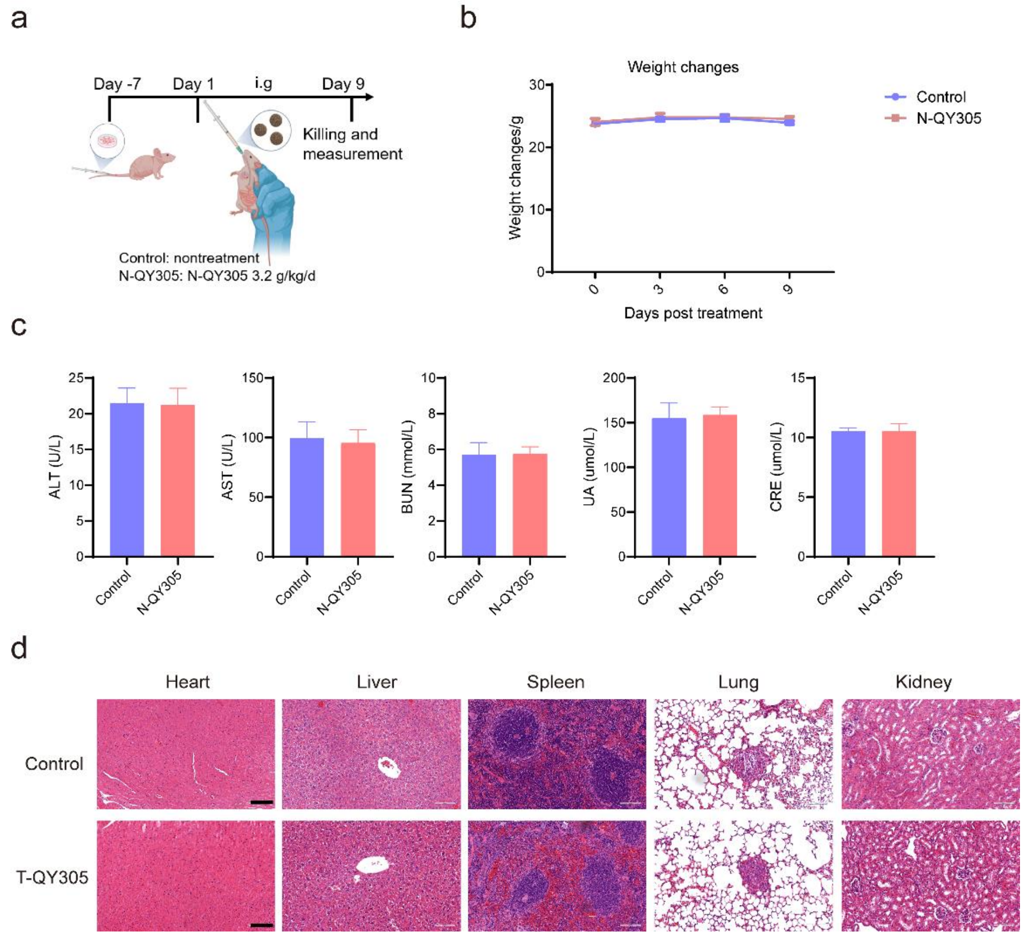

Figure S16. N-QY305 had no toxicity on major organs in balb/c nude mice. **a** Experimental timeline for N-QY305 treatment of balb/c nude mice carrying tumor. **b** The body weight was measured and recorded every 3 days (n≥4). **c** AST, ALT, BUN, UA and CRE levels in blood plasma of each group. **d** H&E staining of histological sections from major organs, including heart, liver, spleen, lung and kidney (n=3), scale bar=100 μm.

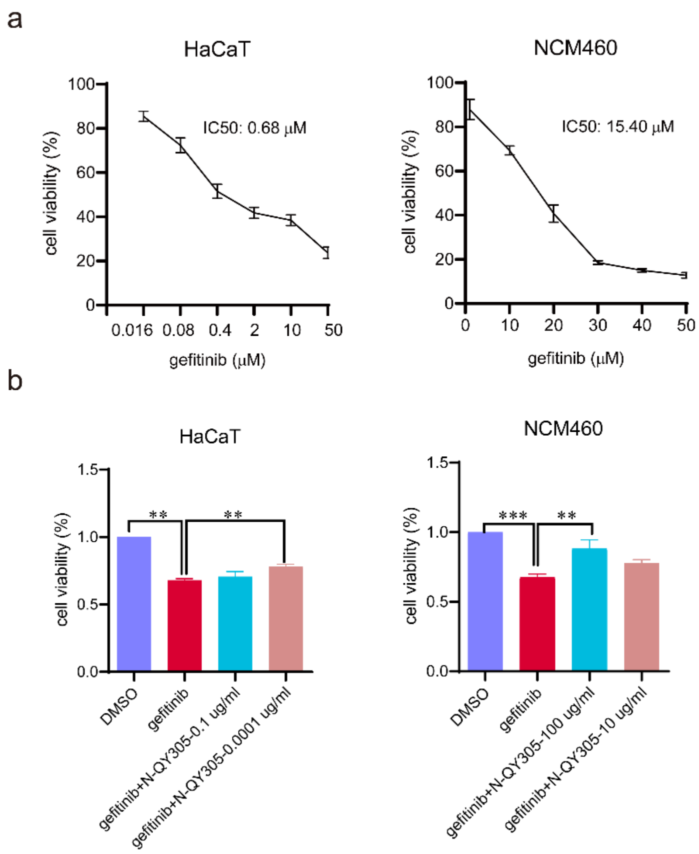

Figure S17. N-QY305 treatment promoted the proliferation of human keratinocytes and colonic epithelial cells *in vitro*. **a** Cell viability of human keratinocytes HaCaT and human colonic epithelial cells NCM460 after treatment with gefitinib for 48 hours. Data are presented as means ± SEM (n=3 replicates). **b** Cell viability of HaCaT cells after treatment with gefitinib (0.03 μM, IC_{80}) combined with N-QY305 (0.0001 μg/mL and 0.1μg/mL) for 48 hours. Cell viability of NCM460 cells after treatment with gefitinib (4.44 μM, IC_{80}) combined with N-QY305 (10 μg/mL and 100 μg/mL) for 48 hours. Data are presented as means ± SEM (n=3 replicates).

Supplementary Tables

Table S1

Characteristics of patient 1		
age	58	
sex	male	
targeted therapy	lunfatini, sintilimab	
disease	secondary lung cancer of renal malignancy	
therapeutic agent	T-QY305	
cutaneous adverse reaction (grade1-3)	pre-treatment	post-preatment
rash	3	2
drying	2	1
pruritus	3	2

Table S2

Characteristics of patient 2

age	60	
sex	female	
targeted therapy	afatinib	
disease	lung adenocarcinoma	
therapeutic agent	T-QY305	
cutaneous adverse reaction (grade1-3)	pre-treatment	post-treatment
rash	1	1
drying	1	1
pruritus	2	1

Table S3

NO.	RT (min)	Identification	Predicted Formula	m/z
1	2.700	Chlorogenic acid	$C_{16}H_{18}O_9$	353.2 [M-H]-
2	4.817	Secoxyloganin	$C_{17}H_{24}O_{11}$	403.2 [M-H]-
3	7.083	Isoliquiritin	$C_{21}H_{22}O_9$	417.1 [M-H]-
4	11.117	Liquiritin	$C_{21}H_{22}O_9$	417.1 [M-H]-
5	16.317	Glycyrrhizinic acide	$C_{42}H_{62}O_{16}$	821.3 [M-H]-

Table S4

NO.	RT (min)	Identification	Predicted Formula	m/z
1	2.483	5-caffeoylquinic acid	$C_{16}H_{18}O_9$	353.2 [M-H]-
2	2.750	Chlorogenic acid	$C_{16}H_{18}O_9$	353.2 [M-H]-
3	3.200	Swertiamarine	$C_{16}H_{22}O_{10}$	373.1 [M-H]-
4	5.633	Secoxyloganin	$C_{17}H_{24}O_{11}$	403.2 [M-H]-
5	6.900	Isoliquiritin	$C_{21}H_{22}O_9$	417.1 [M-H]-
6	8.300	Isochlorogenic acid or isomers	$C_{25}H_{24}O_{12}$	515.1 [M-H]-
7	8.533	Isochlorogenic acid or isomers	$C_{25}H_{24}O_{12}$	515.1 [M-H]-
8	9.633	Isochlorogenic acid or isomers	$C_{25}H_{24}O_{12}$	515.1 [M-H]-
9	10.600	Liguiritigenin-7-O-D-apiosyl-4'-O-D-glucoside	$C_{30}H_{26}O_{13}$	549.2 [M-H]-
10	10.950	Liquiritin	$C_{21}H_{22}O_9$	417.1 [M-H]-

11	11.317	Ononin	$C_{22}H_{22}O_{10}$	475.1 [M+COOH]-
12	12.583	Calycosin	$C_{16}H_{12}O_{5}$	283.1 [M-H]-
13	14.200	22β-acetoxyl licorice saponin G2	$C_{44}H_{64}O_{19}$	895.3 [M-H]-
14	14.550	Licoricesaponin A3	$C_{48}H_{72}O_{21}$	983.3 [M-H]-
15	14.850	22-beta-glycyrrhizinic acide	$C_{44}H_{64}O_{18}$	879.3 [M-H]-
16	15.083	Licoricesaponin G2 isomer	$C_{42}H_{62}O_{17}$	837.3 [M-H]-
17	15.733	Licoricesaponin G2	$C_{42}H_{62}O_{17}$	837.3 [M-H]-
18	16.283	Glycyrrhizinic acide	$C_{42}H_{62}O_{16}$	821.3 [M-H]-
19	16.883	Glycyrrhizinic acide isomer	$C_{42}H_{62}O_{16}$	821.3 [M-H]-
20	17.750	Licoricesaponin B_2	$C_{42}H_{64}O_{15}$	807.3 [M-H]-
21	18.733	Astragaloside I	$C_{45}H_{72}O_{16}$	913.4 [M+COOH]-

Table S5

NO.	RT (min)	Identification	Predicted Formula	m/z
1	2.633	Chlorogenic acid	$C_{16}H_{18}O_9$	352.2 [M-H]-
2	2.900	Loganin or isomers	$C_{17}H_{26}O_{10}$	389.1 [M-H]-
3	3.367	Swertiamarine	$C_{16}H_{22}O_{10}$	373.2 [M-H]-
4	5.683	Secoxyloganin	$C_{17}H_{24}O_{11}$	403.2 [M-H]-
5	6.267	4-Hydroxychalcone-4'-O-glucoside	$C_{21}H_{22}O_{10}$	433 [M-H]-
6	6.800	Rutin	$C_{27}H_{30}O_{16}$	609.1 [M-H]-
7	8.150	Unknown	$C_{27}H_{31}O_{16}$	610.4 [M-H]-
8	8.533	Isochlorogenic acid or isomers	$C_{25}H_{24}O_{12}$	515.1 [M-H]-
9	9.583	Unknown	$C_{48}H_{68}O_5$	723.4 [M-H]-
10	10.033	Aldosecologanin	$C_{34}H_{46}O_{19}$	757.2 [M-H]-
11	10.617	Unknown	$C_{42}H_{61}O_{17}$	836.5 [M-H]-

12	11.467	Unknown	$C_{48}H_{70}O_{19}$	949.6 [M-H]-
13	16.283	Glycyrrhizinic acide	$C_{42}H_{62}O_{16}$	821.3 [M-H]-
